# Ochraceopyronide, a Rare α-Pyrone-C-lyxofuranoside from a Soil-Derived Fungus *Aspergillus ochraceopetaliformis*

**DOI:** 10.3390/molecules26133976

**Published:** 2021-06-29

**Authors:** Mostafa A. Asmaey, Dennis Abatis, Ahmed S. Abdel-Razek, George Lambrinidis, Ioanna Chinou, Nikolas Fokialakis, Nikolaos Tsafantakis, Mohamed Shaaban, Nektarios Aligiannis

**Affiliations:** 1 Division of Pharmacognosy and Natural Products Chemistry, Department of Pharmacy, School of Health Sciences, National and Kapodistrian University of Athens, 15771 Athens, Greece; masmaey1@gmail.com (M.A.A.); ichinou@pharm.uoa.gr (I.C.); fokialakis@pharm.uoa.gr (N.F.); ntsafantakis@pharm.uoa.gr (N.T.); aligiannis@pharm.uoa.gr (N.A.); 2 Department of Chemistry, Faculty of Science, Al-Azhar University, Assiut Branch, Assiut 71524, Egypt; 3 Microbial Chemistry Department, Genetic Engineering and Biotechnology Research Division, National Research Centre, El-Buhouth St. 33, Dokki-Cairo 12622, Egypt; ahmedshukri_sci@yahoo.com; 4 Division of Pharmaceutical Chemistry, Department of Pharmacy, School of Health Sciences, National and Kapodistrian University of Athens, 15771 Athens, Greece; lamprinidis@pharm.uoa.gr; 5 Pharmaceutical and Drug Industries Research Division, Chemistry of Natural Compounds Department, National Research Centre, El-Buhouth St. 33, Dokki-Cairo 12622, Egypt; mshaaba@gmail.com

**Keywords:** *Aspergillus* *ochraceopetaliformis*, α-pyrones, C-lyxofuranosides, sugar conformations, triterpenoids, polyketides, antimicrobial activity

## Abstract

The fungal strain was isolated from a soil sample collected in Giza province, Egypt, and was identified as *Aspergillus* *ochraceopetaliformis* based on phenotypic and genotypic data. The ethyl acetate extract of the fungal strain exhibited promising activity levels against several pathogenic test organisms and through a series of ^1^H NMR guided chromatographic separations, a new α-pyrone-C-lyxofuranoside (**1**) along with four known compounds (**2**–**5**) were isolated. The planar structure of the new metabolite was elucidated by detailed analysis of its 1D/2D NMR and HRMS/IR/UV spectroscopic data, while the relative configuration of the sugar moiety was determined by a combined study of NOESY and coupling constants data, with the aid of theoretical calculations. The structures of the known compounds—isolated for the first time from *A*. *ochraceopetaliformis*—were established by comparison of their spectroscopic data with those in the literature. All isolated fungal metabolites were evaluated for their antibacterial and antifungal activities against six Gram-positive and Gram-negative bacteria as well as against three human pathogenic fungi.

## 1. Introduction

Fungi constitute one of the most abundant kingdoms of living organisms on Earth, with more than 120,000 identified species up to date. The revised estimation of the species yet to be discovered lies in the extraordinary range of 2.2 to 3.8 million [1,2]. The ecological and economic impact of these ubiquitous eukaryotic microbes is enormous and diverse, from the role of yeasts in the fermentation industry and the production of life-saving drugs [3,4] to body infections and the toxification of foodstuffs, livestock, and crops by the mycotoxins [5,6].

Through millions of years of chemical warfare for development and defense, fungi have developed a powerful genetic and biochemical machinery [7], making them a vast source of natural products with unique structures [8,9] and with a wide range of biological activities, including those against bacterial and fungal infections (antibiotics) [10,11].

An important portion of the chemo-diversity of fungi consists of the fungal glycosides, with a variety of structural features on both the aglycone and the sugar parts. The sugar unit plays a crucial role in their antimicrobial, antiviral, and cytotoxic properties [12,13].

*Aspergillus ochraceopetaliformis* is a rare fungal species belonging to the yellow *aspergilli* [14], which was only recently proven to be human pathogenic in a case of a toenail infection [15].

Research on its potential to produce bioactive secondary metabolites is relatively limited. The highly oxygenated α-pyrone merosesquiterpenoids, ochraceopones A−E with antiviral activity and isoasteltoxin, asteltoxin, asteltoxin B and the humulane-derived sesquiterpenoids ochracenes A−I were isolated from an Antarctic soil-derived strain [16,17]. From a Chinese sponge-derived strain, asteltoxin, asteltoxins G, B, C, along with ochratoxins A1, A, B, ochratoxin A methyl ester and ochratoxin B methyl ester were identified [18]. A hexadepsipeptide and its hydrolyzed linear analog were isolated from a Korean marine sediment strain, with moderate/weak enzyme inhibition activities [19]. Polyketides asperochrapyran and asperochralactones A−D together with another 12 known polyketides were found produced by a strain isolated from plant leaves and examined for cytotoxic and anti-inflammatory properties [20].

The ethyl acetate extract of an Indian strain was found to exhibit significant quorum sensing attenuation potential and prevention of biofilm formation of the opportunistic nosocomial pathogen *Pseudomonas aeruginosa* [21]. Finally, in a very recent screening survey, including in vitro antibacterial, antifungal, antioxidant and cytotoxic assays of extracts of several symbiotic fungi, isolated from 10 hard and soft coral species from the Red Sea coral reef in Egypt, the ethyl acetate extract of the strain *A*. *ochraceopetaliformis* MN083316 was shown to be the most active in all assays. From this extract, ditryptophenaline was isolated and suggested to highly contribute to the above activities [22].

**Figure 1:**
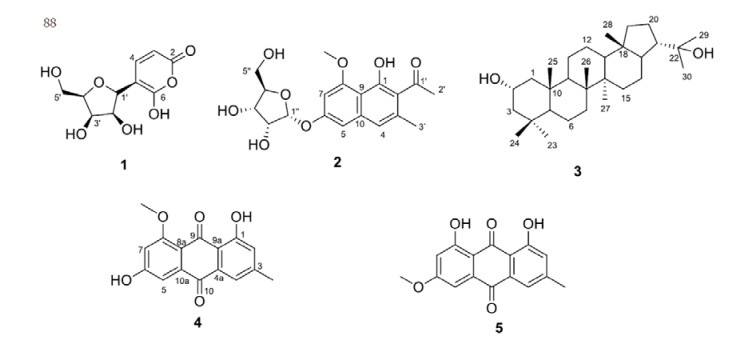
Structures of compounds **1**–**5**.

In the course of our quest for natural products with antibiotic activity [23], we initiated a phytochemical study of the ethyl acetate extract of a soil strain of *Aspergillus ochraceopetaliformis* ASAI, collected in Giza province in Egypt. We present herein the isolation and structure characterization of a rare α-pyrone-*C*-glycoside, Ochraceopyronide (**1**), and 4 known metabolites (Figure 1), isolated for the first time from this species. The antibacterial and antifungal activities of all the obtained compounds were also investigated.

## 2. Results and Discussion

### 2.1. Isolation and Phylogenetic Identification of ASAI

**Figure 2:**
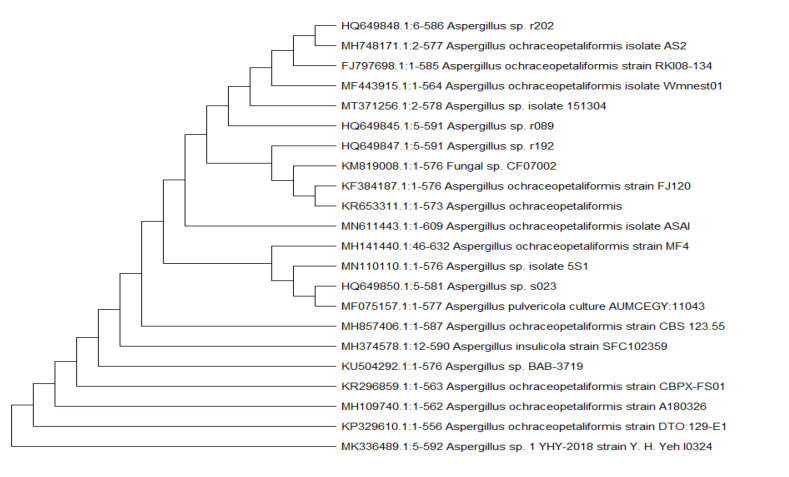
Neighbor-joining phylogenetic tree of the fungal strain ASAI based on 18S rRNA gene sequences, showing its close relationship to *Aspergillus* spp.

The fungal strain ASAI was obtained from a soil sample collected at Giza province, Egypt. The strain was identified based on its 18S rRNA gene sequencing. The genotypic characterization of the sequence, using BLAST at National Centre for Biotechnology Information, confirmed its close similarity to *Aspergillus ochraceopetaliformis* (99–100%). The evolutionary history (Figure 2) was deduced by using the neighbor-joining method. This analysis involved 22 nucleotide sequences. All positions with less than 95% site coverage were eliminated. The gene sequence has been deposited in GenBank database (accession no. MN611443).

### 2.2. Extraction and Purification

The crude EtOAc extract of the fermented solid culture of the fungus *A*. *ochraceopetaliformis* was subjected to column chromatographies on silica gel, Sephadex LH20, followed by preparative TLC for the final purification, resulting in the isolation of five compounds including the α-pyrone-C-glycoside, 6-OH-2-pyrone-5-C-lyxofuranoside, which we named ochraceopyronide (**1**). Among the known metabolites, the polyketide glycoside isotorachrysone 6-*O*-*α*-d-ribofuranoside (**2**) [24], the triterpene 2-hydroxydiplopterol (**3**) [25] and the two anthraquinone derivatives physcion (**4**) [26] and questin (**5**) [27] are included (Figure 1).

### 2.3. Structure Elucidation

Ochraceopyronide (**1**) was isolated as a dark brown, optically active, amorphous solid. Its molecular formula C_10_H_12_O_7_ was determined based on the (+)HR-ESI-MS of its pseudomolecular ion peak [M + H]^+^ at *m/z* 245.0663 amu (calculated for [C_10_H_13_O_7]_]^+^, 245.0656 amu), a formula indicating five degrees of unsaturation (five DBEs). The IR spectrum exhibited absorption bands for hydrogen-bonded hydroxyl groups at 3466 cm^−1^ (broad), along with indicative bands for α,β unsaturated ester, or lactone carbonyl moiety (1637, 1695 cm^−1^) and bending vibration of aromatic O-H at *ν* 1384 cm^−1^. The UV absorption bands at λ_max_ 218 and 262 nm supported the presence of an α,β unsaturated carbonyl in the structure.

In the ^1^H NMR spectrum of **1** in DMSO-*d*_6_ (Table 1), two individual groups of proton signals were observed, accounting for two isolated spin systems according to the ^1^H-^1^H COSY spectrum. The first spin system consisted of three oxymethine protons at *δ_H_* 4.00 (H-2′), 3.90 (H-3′), 3.73 (H-4′), two oxymethylene protons at *δ_H_* 3.58 (H-5a′) and 3.62 (H-5b′), in addition to a downfield-shifted oxymethine doublet at *δ_H_* 6.00 ppm. Within the same spin system, the signals of three hydroxyl protons were detected resonating at *δ_H_* 5.56, 5.43 and 5.01, as broad singlets, displaying weak but discernible COSY correlations with their corresponding oxymethine protons H-2′, H-3′ and the one of the oxymethylene (H-5b′), respectively. These data suggested the presence of a pentofuranoside moiety in the molecule with the oxymethine proton doublet at *δ_H_* 6.00 being the anomeric proton.

**Figure 3:**
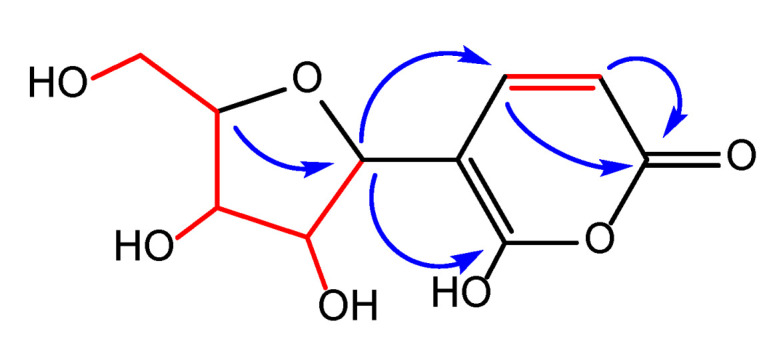
^1^H-^1^H COSY (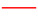) and selected key HMBC (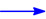) correlations for Ochraceopyronide.

The anomeric H-1′ was attached to a carbon resonating at *δ_C_* 85.1 ppm. The high field chemical shift of the anomeric carbon was indicative of a furanose rather than a pyranose sugar ring, connecting with a C- instead of an O-glycosidic bond to the aglycon moiety [28,29,30,31,32]. The furanose nature of the sugar ring was further supported by a weak but diagnostic HMBC cross peak from H-4′ to the anomeric carbon C-1′ [33] (Figure 3).

The second spin system consisted of two proton signals. We detected two olefinic methines of a double bond with a *cis* geometry at *δ_H_* 7.62 (H-4, d, *J* = 8.1 Hz, C-4 at *δ_C_* 142.3 ppm) and 5.55 (H-3, d, *J* = 8.1 Hz, C-3 at *δ_C_* 100.0 ppm). The last proton signal we observed was the one of an isolated aromatic or enolic hydroxyl proton at *δ_H_* 11.22. This hydroxyl group was attached to a carbon resonating at *δ_C_* 150.5 ppm.

The downfield shifts of H-4/C-4 can be well explained when the double bond is conjugated to an ester or lactone carbonyl which was secured by the HMBC correlations of both H-4 and H-3 with a carbonyl moiety at *δ_C_* 163.4 (C-2) (Figure 3).

By taking into account the above NMR data, the rest of the ^1^H-^1^H-COSY and HMBC correlations (Table 1) and the five DBEs of the molecular formula, we concluded the gross structure of (**1**) as depicted in Figure 1.

**Figure 4:**
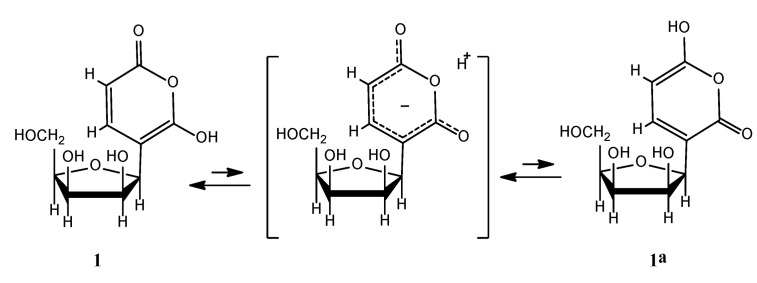
Tautomerization in Ochraceopyronide (**1**).

The extended π-conjugation, which takes place in the 6-OH-*α*-pyrone ring, incorporating the enol-keto equilibrium, probably accounts for the unusual downfield shift of the anomeric proton H-1′. In our case, because of a clear HMBC cross-peak between H-1′ and C-6, the most abundant tautomer of (**1**) in DMSO-*d*_6_ solution at 25 °C, corresponds to (**1**) rather than to (**1a**) (Figure 4).

Surprisingly, we detected only nine carbon signals in the ^13^C NMR spectrum, with the missing one being C-5 of the 6-OH-α-pyrone ring. The same phenomenon had been reported in 2001 for Tetillypyrone and nor-Tetillyapyrone, two 5-C-(2′-deoxy)-furanosyl-6-hydroxyl-α-pyrones isolated from a sponge collected in Thailand waters [34].

The authors initially attributed the lack of observation, in both compounds, of a distinct signal of the same carbon C-5 in the ^13^C NMR spectrum to the coincidental superimposition of the signals of C-5 and C-3, even though C-3 is a *sp^2^* methine in nor-Tetillyapyrone and a *sp^2^* quaternary carbon in Tetillyapyrone, with quite different chemical environments. In 2007, the same authors ruled out the possibility of the coincidental superimposition, without providing any further explanation for the missing carbon C-5 [35].

Assuming that the invisibility of the C-5 resonance in the ^13^C NMR spectrum of (**1**) at 25 °C is due to the splitting of its signal into several ones, corresponding to different conformers generated by the relatively hindered rotation through the C-1′-C-5 glycosidic bond, we ran ^13^C NMR experiments at elevated temperatures in DMSO-*d*_6_ (Supplementary Materials) to facilitate the rotation and obtain the average resonance of C-5 [36,37]. Unfortunately, we were not able to detect any average carbon signal for C-5, even at 80 °C.

Concerning the relative configuration of the furanose sugar moiety, we utilized the results from the NOESY experiment, combined with the ^3^*J* coupling constants data and theoretical calculations.

Five membered tetrahydrofuran rings are known to adopt in solution several puckered conformations from a sum of 10 Envelope (E) and 10 Twisted (T) combinations. These conformers interconvert through pseudo-rotation and their stability/abundancy depends on the substituents, the solvent, and the temperature [38,39]. In contrast to the six-membered pyranose rings in which the assignment of the stereochemistry of the sugar carbons can more easily be relied on the spin–spin coupling constants of their vicinal protons, due to the predominance of one of usually two stable, rigid conformers (^4^C_1_ or ^1^C_4_), in furanose sugars, the flexibility of the ring prevents the direct correlation of the magnitude of the ^3^*J*_H-H_ values with the *cis* or *trans* geometry of the vicinal protons.

**Figure 5:**
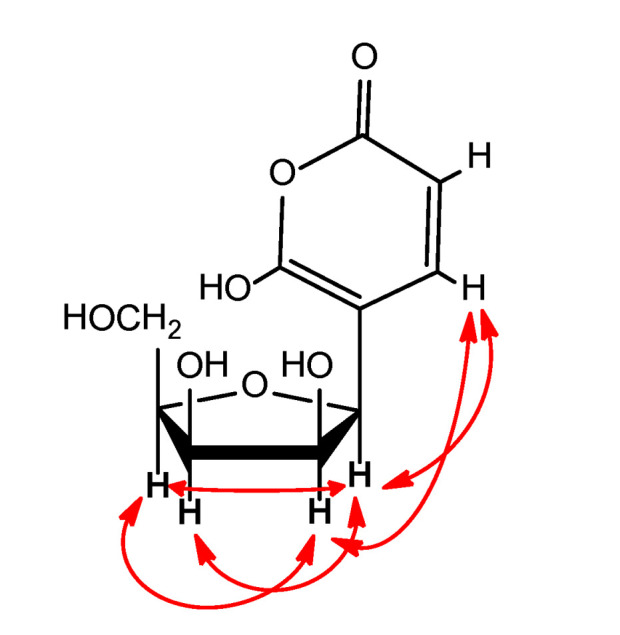
Key NOE correlations in Ochraceopyronide (**1**).

**Figure 6:**
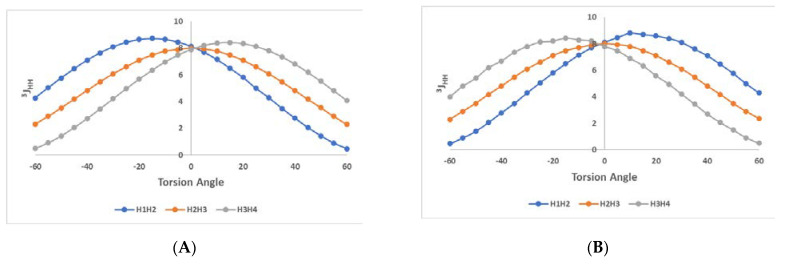

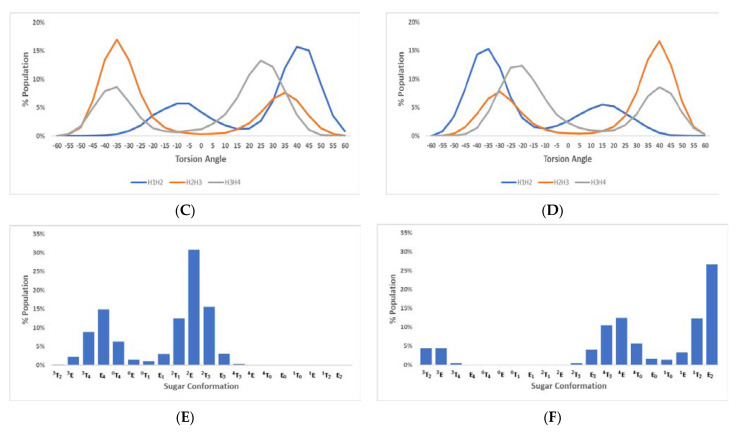
(**A**,**B**) Theoretical ^3^*J*_HH_ coupling constants distribution; (**C**,**D**) distribution of ^3^*J*_HH_ coupling after MD; (**E**,**F**) distribution of furanose conformers of *β*-d- and *β*-l-c-lyxofuranoside residues of Ochraceopyronide (**1**), respectively.

The observed splitting patterns of the furanose oxymethines are the weighted averages of those of each one of all the existing conformers in solution. This is the reason why the relative configuration in furanosides has been based mainly on NOESY or ROESY data [38,39,40]. In the NOE spectrum of (**1**), two sets of cross-peaks secured its relative stereochemistry, providing important information concerning the inherent conformational flexibility of the five-membered sugar ring and the rotation around the C-glycosidic bond C1‘-C5. In the first one, we detected the correlations of the anomeric proton H-1′ with H-4′, H-3′ and H-2′, while the second one included the correlations of H-4 of the pyrone ring with H-1′, H-2′ and H-3′. (Figure 5)**.** Due to the homofacial stereo-relation of all the furanose oxymethines, the C-glycosidic bond is *β*, thus allowing the development of multiple intramolecular hydrogen bonds between the sugar OH-groups and the enolic 3-OH. This in turn, could explain not only the shift of the enol-keto equilibrium of the α-pyrone ring in favor of (**1**), but also the stabilization of several preferred conformers (Figure 6) that could split the ^13^C signal of C-5, thus accounting for its invisibility in the ^13^C NMR spectrum, even at higher temperatures.

Based on the above results, the sugar residue of (**1**) is, therefore, a *β*-d-C-lyxofuranoside or its enantiomer *β*-l-C-lyxofuranoside.

In two relatively recent articles involving coupling constant computational studies in five-membered homo and heterocycles, an unequivocal relation between ^3^*J*_H-H_ and relative configuration has been proposed, although confined to *trans* vicinal protons [33,41]. The authors found a ^3^*J*_H-H_ threshold of 2 Hz for H-1/H-2 and H-3/H-4 and a threshold of 3.5 Hz for H-2/H-3, under which the geometry of the above vicinal proton pairs should always be *trans*. In these theoretical calculations, the model compounds that were used were the methoxy-α/β-d-furanosides (ribo-, xylo-, arabino-, lyxo-) or the methyl/methoxy oxolanes, while for the measurements for the dihedral angles and the ^3^*J*_H-H_’s, only the E conformers of the furanoside/oxolane rings were considered.

In **1,** none of the ^3^*J*_H-H_ of the oxymethines H-1′ (d, *J* = 4.5 Hz), H-2′ (br.t, *J* = 3.6 Hz), H-3′ (br.t, *J* = 3.7 Hz), H-4′ (br.q, *J* = 4.6 Hz) that we measured in DMSO-*d*_6_ (25 °C) were lower than 3.5 Hz, in alignment with the conclusions of the previously published theoretical work. We also undertook a computational study to calculate the dihedral angles of the furanose ring and the corresponding vicinal oxymethine coupling constants of the theoretically more abundant conformations, detecting any impact of the α-pyrone ring, and compared them with our experimental ^3^*J*_H-H_ and NOE correlations.

Initially, we designed the *β*-l and *β*-d enantiomers of Ochraceopyronide (**1**) using the Maestro Software (Schrödinger Release 2020-2: Maestro, Schrödinger, LLC, New York, NY, 2020). We generated all possible conformations of the furanose ring based on the pseudorotation equation for torsion angles by Altona and Sundaralingam [38,39], resulting in 20 conformers, (10E and 10T) for each enantiomer (Supplementary Materials). Next, each conformer was solvated on a periodic box with explicit TIP3P water molecules. The GLYCAM_06 [42] and GAFF2 forcefield [43] were applied and we ran 10ns of a molecular dynamic (MD) simulation with AMBER18 software [44]. We analyzed the resulting trajectories by identifying the furanose ring puckering and the variation of all torsion angles between H1′-C1′-C2′-H2′, H2′-C2′-C3′-H3′ and H3′-C3′-C4′-H4′. Although we started from all possible E and T conformations, the D enantiomer was mainly populated from ^3^E to ^0^T_4_ and E_1_ to E_3_ conformers (Figure 6E), while the L enantiomer was mainly populated from ^3^T_2_ to ^3^E, E_3_ to ^4^T_0_ and ^1^E to E_2_ conformers (Figure 6F). The α-pyrone substituent seems to disturb the free conformation change on the furanose ring. Furthermore, we explored the theoretical ^3^*J* coupling of H1′-H2′, H2′-H3′ and H3′-H4′ by calculating the torsion angles *Φ* of H1′-C1′-C2′-H2′, H2′-C2′-C3′-H3′ and H3′-C3′-C4′-H4′, respectively. All torsion angles were within the range of −60° to + 60°, as expected for all vicinal protons being *cis*-oriented. Each torsion angle *Φ* passed through the Altona Equation to predict the theoretical ^3^*J*_HH_ coupling constant. The deviation of each torsion angle was ± 20° for each unique sugar conformation, leading to ± 2 Hz deviation in ^3^*J*_HH_ prediction. Using the appropriate substituents on carbon atoms C1′, C2′ C3′ and C4′, the theoretical ^3^*J*_HH_ values were calculated [45] and presented in Figure 6A for the d-enantiomer and Figure 6B for the l-enantiomer. The distribution of each torsion angle *Φ* is depicted in Figure 6C and Figure 6D for d- and l- enantiomer, respectively.

***β*-d-C-lyxofuranoside residue of 1.** For ^3^*J*_H1-H2_, the majority of conformers reside on *Φ* = 30 to 50 (^3^*J* = 4.3–1.4) and *Φ* = −20 to 5 (^3^*J* = 7.7–8.7); for ^3^*J*_H2-H3_, on *Φ* = −20 to −50 (7.1–3.5 Hz) and *Φ =* 20 to 50 (7.1–3.6 Hz); and for ^3^*J*_H3-H4_, on *Φ* = −30 to −50 (4.2–1.4 Hz) and *Φ* = 10 to 40 (8.4–6.8 Hz).

***β*-l-C-lyxofuranoside residue of 1.** For ^3^*J*_H1-H2_, the majority of conformers reside on *Φ* = 0 to 30 (8.1–8.8 Hz) and *Φ* = −20 to −50 (5.8–1.4 Hz); for ^3^*J*_H2-H3_, on *Φ* = −20 to −40 (7.1–4.8 Hz) and *Φ =* 25 to 55 (6.6–2.9 Hz); and for ^3^*J*_H3-H4_, on *Φ* = −10 to −40 (8.3–6.7 Hz) and *Φ* = 30 to 50 (4.2–1.5 Hz).

The above results show that only a combination of several existing conformers in solution for **1,** with different abundancies/stabilities, would account for a better proximity between theoretical and experimental ^3^*J*_H-H_ values. This could also explain the numerous correlations we observed in the NOE spectrum between protons that are spatially close to each other in some conformations but more distant in others.

**Figure 7:**
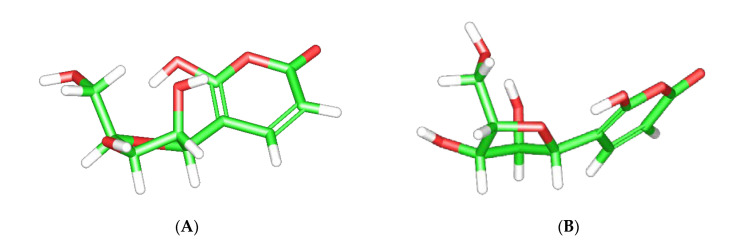
Representative conformers (^2^E for *β*-d-C-lyxofuranoside (**A**) and ^2^T_3_ for *β*-l-C-lyxofuranoside (**B**)) for the two enantiomers of Ochraceopyronide (**1**).

In Figure 7A, we present a representative theoretical conformer for *β*-d-C-lyxofuranoside residue of **1** (^2^E), and in Figure 7B, a representative theoretical conformer for *β*-l-C-lyxofuranoside residue of **1** (^2^T_3_). Both belong to the group of theoretical conformers with the closest values of ^3^*J*_H-H_ with the experimental ones. The torsion angles measured for each torsion angle in interest are in the range of ± 20°of the mean value presented in Table S3.

To the best of our knowledge, this is the first C-lyxofuranoside isolated from nature. Lyxosides, in general, are rare as natural products, encountered only as *O*-pyranosides [12,46,47], while there are some synthetic works including *O*-lyxofuranosides or *O*-lyxopyranosides as intermediates or targets for finding routes to interconvert sugars [48,49] or for theoretical NMR and physicochemical calculation purposes [50,51,52].

### 2.4. Antimicrobial Activity

The isolated compounds (**1**–**5**) were tested for their antimicrobial activity against two Gram-positive bacteria: *Staphylococcus aureus* and *Staphylococcus epidermidis*; four Gram-negative bacteria: *Escherichia coli*, *Enterobacter cloacae*, *Klebsiella pneumoniae,* and *Pseudomonas aeruginosa*; and three pathogenic fungi: *Candida albicans*, *Candida tropicalis* and *Candida glabrata* (Table 2). The antimicrobial studies showed that ochraceopyronide (**1**), questin (**4**), and physcion (**5**) exerted a weak activity against the Gram-positive bacteria and weak activity or inactivity against the tested Gram-negative bacteria and the human pathogenic fungi. It is noteworthy that compounds **2** and **3** exhibited the strongest antibacterial and antifungal activity (minimal inhibition concentration (MIC) values 0.09–0.87 mg/mL) among assayed metabolites.

## 3. Materials and Methods

### 3.1. Instrumentation

UV spectra were recorded on a UV-2600 spectrometer (Shimadzu, Tokyo, Japan). IR spectra were measured on an IR Affinity-1 spectrometer (Shimadzu). NMR experiments (both one- and two-dimensional spectra) were performed using Bruker Avance 600 MHz ^1^H (150MHz ^13^C) and 400MHz ^1^H (100MHz ^13^C) spectrometers. NMR spectra were acquired in CDCl_3_ (*δ_H_* 7.24 and *δc* 77.0), CD_3_OD (*δ_H_* 3.31 ppm and *δ_C_* 49.0) and DMSO (*δ_H_* 2.50 and *δ_C_* 39.5) and run at 25 °C, except for those in higher temperature in DMSO-*d*_6_. High-resolution mass spectra were obtained in a Q-Exactive Orbitrap platform (Thermo Fisher Scientific, San Jose, CA, USA) connected to a Dionex Ultimate 3000 UHPLC system (Thermo Scientific™Dionex™, Sunnyvale, CA, USA). A Hypersil Gold UPLC C18 (2.1 × 150 mm, 1.9 μm) column (Thermo Fisher Scientific, San Jose, CA, USA) was used. Silica-gel 60 F254 aluminum plates (Merck, Germany) were used for thin-layer chromatography (TLC) to detect compounds of interest. For visualization of separated compounds on TLC-plates, a vanillin spray reagent was used (5.0 g vanillic acid in 100 mL MeOH mixed with 100 mL of 5% *v/v* sulphuric acid in MeOH). Size exclusion chromatography was implemented on Sephadex LH-20 (Sigma, St. Louis, MO, USA).

### 3.2. Isolation and Identification of the Producing Strain

The fungal strain was obtained from a soil sample collected in Giza province, Egypt. The sediment sample was suspended in sterile water and incubated into a reciprocal water bath at 30 °C for 30 min, and then the samples were serially diluted under aseptic conditions. A measure of 100 µL from each dilution were plated on PDA medium (g·L^−1^: Potato infusion, 200 g; Dextrose, 20 g and 20.0 g Agar and 1000 mL of 50% seawater, and pH 7.2). The plates were then incubated at 35 °C for six to eight weeks. The strain was isolated, transferred onto freshly prepared solid media, and deposited in a refrigerator at 4 °C at the Microbial Chemistry Department, National Research Centre (NRC), Egypt until use.

The fungal strain ASAI was identified using molecular protocol by DNA amplification and sequencing of the internal transcribed spacer (ITS) region [53]. The DNA concentration was 100 ng/µL, with a volume of 20 µL. The PCR product was detected by agarose gel and visualized by (UV) fluorescence after ethidium bromide staining [54]. The results of 18S rRNA sequence were compared to the available database at GenBank using BLAST software (blastn) on National Centre Biotechnology Information (NCBI). The phylogenetic tree was constructed using the neighbor-joining tree method [55]. Based on the 18S rRNA gene sequence and phylogenetic data, the strain ASAI was taxonomically identified as *Aspergillus ochraceopetaliformis* (accession no. MN611443). The evolutionary analyses were conducted in MEGA X [56].

### 3.3. Up-Scale Fermentation and Extraction

The spore suspension of the selected strain ASAI was inoculated into 100 mL of ISP2 medium composition (g L^−1^): malt extract, 10; yeast extract, 4; and glucose, 4 dist. H_2_O and incubated at 30 °C for three days as the seed culture. Five milliliters of previously prepared seed culture were used to inoculate 5× 1 L sterilized Erlenmeyer flasks (five flasks) containing rice medium composition: 100 g commercial rice; 150 mL of 50% seawater. The flasks were incubated for 14 days at 35 °C [57]. After harvesting, the culture was mixed with methanol and filtered under a vacuum. The filtered aqueous methanol was concentrated in vacuo, and the remaining water residue was re-extracted by ethyl acetate [58] and then concentrated to dryness to afford 5.5 g of a dark green crude extract.

### 3.4. Isolation and Purification of Secondary Metabolites

The crude extract (5.5 g) was subjected to column chromatography on silica gel (100 × 5 cm, 150 g of silica) and gradually eluted with mixtures of increasing polarity of cyclohexane–CH_2_Cl_2_ and then with CH_2_Cl_2_–MeOH. Guided by TLC/NMR profiling and after pooling, 12 fractions were obtained: F1(617.5 mg), F2(805.0 mg), F3(257.6 mg), F4(901.5 mg), F5(383.0 mg), F6(104.2 mg), F7(132.0 mg), F8(500.0 mg), F9(359.7 mg), F10(900.0 mg), F11(61.0 mg), F12(410.0 mg).

F1 contained mainly fats, while column chromatography of F7 on an open silica column using as mobile phase mixtures of c-Hex/EtOAc (100–50/50) afforded pure compound **3** (75.0 mg). Compound **5** (20.0 mg) was isolated from F2 after washing with cyclohexane. F8 was chromatographed on silica column (DCM/MeOH 100–80/20) to produce F8a and F8b. F8a was further chromatographed using semipreparative HPLC C-8 (H_2_O/MeOH gradient, 65/35 to 5/95 in 55 min) to give compound **4** (2.2 mg, *t_R_* = 22.1). F10 was subjected to another silica column eluted with DCM/MeOH (100–80/20) to afford subfractions F2a and F2b. Further purification of F2b using pTLC afforded **2** (5.7 mg). F11 after defatting with cyclohexane gave pure compound **1** (27.0 mg).

Compounds **2**–**5** were isolated for the first time from *Aspergillus ochraceopetaliformis* and identified as isotorachrysone-6-O-α-d-ribofuranoside (**2**), 2-hydroxydiplopterol (**3**), questin (**4**) and physcion (**5**), by interpretation of their NMR data and comparison with the literature.

***Ochraceopyronide*** (**1**) Dark brown amorphous solid, [*α*]_D_^20^ = +100 (*c* 0.2 mg/mL, DMSO); TLC visualization spot at 254 nm which turned white after spraying with vanillin/sulphuric acid and heating; *R*_f_ = 0.76 (MeOH/H_2_O 50%); UV (MeOH) λ_max_ (log ε): 218 nm (1.52), 262 nm (0.579); IR (pellets KBr) (cm^−1^): 1049, 1120, 1276, 1384, 1466, 1618, 1637, 2924, 3415, 3466, 3541; (+)-HR-ESI-MS: *m/z* 245.0663 (Calc. 245.0656 [M + H]+ for [C_10_H_13_O_7_]^+^); ^1^H NMR/^13^C NMR (600 MHz/150 MHz, DMSO-*d*_6_), see Table 1.

### 3.5. Antimicrobial Assay Using Agar Diffusion–Dilution Test

Based on paper-disk diffusion and dilution assay [59,60], the antimicrobial activity of the isolated compounds was tested against two Gram-positive bacteria: *S. aureus* (ATCC 25923) and *S. epidermidis* (ATCC 12228); four Gram-negative bacteria: *E. coli* (ATCC 25922), *E. cloacae* (ATCC 13047), *K. pneumoniae* (ATCC 13883) and *P. aeruginosa* (ATCC 227853); and three pathogenic fungi: *C. albicans* (ATCC 10231), *C. tropicalis* (ATCC 13801) and *C. glabrata* (ATCC 28838).

The antimicrobial activity of the isolated compounds were further determined using the agar dilution technique [23]. For all the assays, stock solutions of the tested samples were prepared at 10 mg/mL concentration. Serial dilutions of the stock solutions in broth medium (100 μL of Muller-Hinton broth or on Sabouraud broth for the fungi) were prepared in a microtiter plate (96 wells). Then, 1 μL of the microbial suspension (the inoculum, in sterile distilled water) was added to each well. For each strain, the growth conditions and the sterility of the medium were checked, then the plates were incubated as referred above. MICs were determined as the lowest concentrations preventing visible growth. Standard antibiotic netilmicin and amoxicillin (at concentrations of 4 to 88 μg/mL) were used to control the sensitivity of the tested bacteria, with amphotericin B and 5-fluocytocine (at concentrations of 0.4 to 1 μg/mL) as a control against the tested fungi (Sanofi, Diagnostics Pasteur at concentrations of 30, 15 and 10 μg/mL). For each experiment, any pure solvent used was also applied as a blind control. The experiments were repeated three times and the results were expressed as average values.

### 3.6. Molecular Dynamic Simulations

MD simulations were run utilizing Amber18 and AmberTools18. The global minimum structure from conformation search was passed to leap software (AmberTools19). Gaff2 and Glycam-06EPb force fields were utilized as implemented on Amber18. The compound was solvated inside a periodic box (45.9Å × 61.6Å × 39.5Å) with 1839 TIP3P water molecules. The system was heated from 0 to 300 K using 2500 steps of 0.002 fs and the equilibrated structure was further simulated for 12.5 ns on 300K. A total of 1250 conformers were saved and further clustered to conclude the most dominant furanose ring conformations.

## 4. Conclusions

A new *α*-pyrone-C-lyxofuranoside was isolated from an Egyptian strain of the phytochemically underexplored fungus *Aspergillus ochraceopetaliformis*, which we named Ochraceopyronide (1). As a natural product, ochraceopyronide contains three rare structural characteristics combined; the sugar moiety is a lyxose, its cyclization is of the furanose form, and it is connected to the aglycon by a C-glycosidic bond. The carbon atom C-5 of the aglycon 6-OH-2-pyrone, to which the lyxofuranose is connected, was invisible in the ^13^C NMR, probably due to the relatively hindered rotation through the C-glycosidic bond. Our efforts to observe the missing C-5 resonance by running the ^13^C NMR of ochraceopyronide at higher temperatures were unsuccessful. The molecular dynamics simulation that was performed, starting from all possible conformers of ochraceopyronide, confirmed the inherent flexibility of the 5-membered lyxofuranose ring and the effect of the α-pyrone aglycon on its torsion angles and the values of the ^3^*J* coupling constants of the oxymethines. Ochraceopyronide exists in solution in an equilibrium of several conformers of different abundancies and the experimental ^3^*J*‘s of the oxymethines are the weighted averages of those of each one of all the existing conformers. Along with ochraceopyronide, four known compounds were isolated (**2**–**5**) for the first time from this species. The antimicrobial activity of the five isolated compounds was evaluated against six bacterial strains and three pathogenic fungi. Compounds **2** and **3** exhibited the strongest antibacterial and antifungal activity with minimal inhibition concentration values (MIC) from 0.09 to 0.87 mg/mL.

## Figures and Tables

**Table 1:** NMR data of **1** (600 MHz, DMSO-*d*_6_, *δ* ppm) ^a,b^.

Position	^1^H (*J* in Hz)	^13^C	COSY	HMBC	NOESY
2	-	163.4	-	-	-
3	5.55, d (8.1)	100.0	H-4	C-2, C-4	H-4
4	7.62, d (8.1)	142.3	H-3	C-2, C-3, C-6, C-1′	H-3, H-1′, H-2′(w), H-3′, H-5a’, H-5b’
5	-	n.d	-	-	-
6	-	150.5	-	-	-
6-OH	11.22, br.s	-	-	-	H-2′, H-3′
1′	6.00, d (4.5)	85.1	H-2′	C-4, C-6, C-2′	H-4, H 2′, H 3′(w), H-4′
2′	4.00, br.t (3.6)	75.2	H-1′, H-3′	C-1′, C-3′, C-4′	H-1′, H 3′, H-4′, 2′-OH, 3′-OH, 5′-OH (w)
2′-OH	5.56, ovlp.	-	H-2′	-	H-2′, H-3′
3′	3.90, br.t (3.7)	75.5	H-2′, H-4′	C-1′, C-2′, C-4′, C-5′	H-2′,H-1′,H-4′,H-4, H-5a,b′ 2′-OH, 3′-OH, 5′-OH
3′-OH	5.43, br.s	-	H-3′	-	H-2′, H-3′
4′	3.73, br.q (4.6)	84.7	H-5′a, H-5′b	C-1′, C-3′, C-5′	H-1′, H-2′, H-3′, H-5′a,b
5′a 5′b	3.58, dd (4.6, 11.5)3.62, dd (5.6, 11.5)	60.7	H-4′	C-3′, C-4′	H-4, H-3′, H-4′
5′-OH	5.01, br.s	-	H-5a’,H5b’	-	H-2′, H-3′

^a 1^H chemical shifts referenced to residual proton (CD_2_H)SO(CD_3_) at *δ_H_* 2.50, followed by multiplicity and coupling constants. ^13^C chemical shifts referenced to (^13^CD_3_)SO(CD_3_) at *δc* 39.5. ^b^ Assignments were based on HSQC-DEPT, ^1^H-^1^H COSY and HMBC experiments recorded in DMSO-*d*_6_. nd: not detected.

**Table 2:** Antimicrobial activity of the isolated compounds (**1**–**5**) (zones of inhibition/MIC mg/mL, *n* = 3).

	*S. aureus*	*S. epidermidis*	*P. aeruginosa*	*K. pneumoniae*	*E. cloacae*	*E.coli*	*C. albicans*	*C. tropicalis*	*C. glabrata*
1	9/1.19	9/1.22	na	na	na	na	na	na	na
2	16/0.10	17/0.09	12/0.45	13/0.50	13/0.47	13/0.42	11	12/0.90	13/0.72
3	15/0.23	16/0.25	12/0.52	13/0.61	13/0.65	13/0.47	12/0.87	12/0.82	12/0.7 8
4	8/1.22	9/1.15	na	na	na	na	na	na	na
5	10/1.35	9/1.25	8/1.62	na	8/1.97	8/1.90	9	10	10
Netilmicin	20/0.2 × 10^−3^	26	21	24	21	23	nt	nt	nt
Amoxicillin	22	24	25	23	22	22	nt	nt	nt
5-fluocytocin	nt	nt	nt	nt	nt	nt	22	22	24
AmphotericinB	nt	nt	nt	nt	nt	nt	24	24	25

na: not active, nt: not tested.

## Data Availability

Data is contained within the article or Supplementary Materials.

## Supplementary Materials

TS1–TS5: Tables of NMR data of **1**–**5**, TS6: Antimicrobial activities of the crude fungal extract, FS1–FS9: HR-MS, UV, IR, 1D and 2D NMR spectra of **1** in DMSO-*d*_6_, FS10–FS13: ^1^H NMR & ^13^C NMR of **1** in DMSO-*d*_6_ at elevated temperatures, FS14–FS16: 1D and 2D NMR spectra of **1** in MeOD-*d*_4_, FS17–FS26: 1D and 2D NMR spectra of **2**–**5**, FS27–FS30: Tables and diagrams of theoretical calculations and distributions of conformers of **1**.

## Sample Availability

Not available.
