# Supplementary material for: Ochraceopyronide, a Rare α-Pyrone-C-lyxofuranoside from a Soil-Derived Fungus Aspergillus ochraceopetaliformis
Source: Molecules. 2021 Jun 29;26(13):3976. doi: 10.3390/molecules26133976 (PMC8271807; doi:10.3390/molecules26133976)

Supplementary Materials

Ochraceopyronide, a rare α-pyrone-C-lyxofuranoside from a soil-derived fungus *Aspergillus ochraceopetaliformis*

Mostafa A. Asmaey[1,2], Dennis Abatis[1*], Ahmed S. Abdel-Razek[3], George Lambrinidis[4], Ioanna Chinou[1], Nikolas Fokialakis[1], Nikolaos Tsafantakis[1], Mohamed Shaaban[5] and Nektarios Aligiannis[1]

[1] Division of Pharmacognosy and Natural Products Chemistry, Department of Pharmacy, School of Health Sciences, National and Kapodistrian University of Athens, 15771, Athens, Greece.

[2] Department of Chemistry, Faculty of Science, Al-Azhar University, Assiut Branch, 71524, Assiut, Egypt.

[3] Microbial Chemistry Department, Genetic Engineering and Biotechnology Research Division, National Research Centre, El-Buhouth St. 33, Dokki-Cairo 12622, Egypt.

[4] Division of Pharmaceutical chemistry, Department of Pharmacy, School of Health Sciences, National and Kapodistrian University of Athens, 15771, Athens, Greece.

[5] Chemistry of Natural Compounds Department, Pharmaceutical and Drug Industries Research Division, National Research Centre, El-Buhouth St. 33, Dokki-Cairo 12622, Egypt.

Contents

Table S1: NMR data of Ochraceopyronide (**1**) in MeOD (600 MHz, *δ* ppm) [a].. 4

Table S2: NMR data of isotorachrysone 6-O-α-D-ribofuranoside (**2**) (400 MHz, *δ* ppm)[a].............................. 5

Table S3: NMR data of 2-OH-diplopterol (**3**) (400 MHz, *δ* ppm)[a]..6

Table S4 : NMR data of questin (**4**) (400 MHz, *δ* ppm)[a]..7

Table S5 : NMR data of physcion (**5**) (400 MHz, *δ* ppm)[a]... 8

Table S6 : Antimicrobial activities of the crude fungal extract in agar diffusion assays (mm diameter).........8

Figure S1: HR-ESI-MS spectrum of compound Ochraceopyronide (**1**)... 9

Figure S2: IR spectrum of Ochraceopyronide (**1**) (KBr disc)...10

Figure S3: UV spectrum of Ochraceopyronide (**1**) in MeOH...11

Figure S4: ^{1}H NMR spectrum of Ochraceopyronide (**1**) recorded in (DMSO-d_6) at 600 MHz........................ 12

Figure S5: ^{13}C NMR spectrum of Ochraceopyronide (**1**) recorded in (DMSO-d_6) at 150 MHz...................... 13

Figure S6: ^{1}H-^{1}H COSY spectrum of Ochraceopyronide (**1**) recorded in (DMSO-d_6) at 600 MHz.................. 14

Figure S7: HSQC DEPT spectrum of Ochraceopyronide (**1**) recorded in (DMSO-d_6) at 600 MHz.................... 15

Figure S8: HMBC spectrum of Ochraceopyronide (**1**) recorded in (DMSO-d6) at 600 MHz............................16

Figure S9: NOESY spectrum of Ochraceopyronide (**1**) recorded in (DMSO--d_6) at 600 MHz......................... 17

Figure S10: ^{1}H NMR spectrum of Ochraceopyronide (**1**) recorded in (DMSO-d_6) at 400 MHz at 40 °C......... 18

Figure S11: ^{1}H NMR spectrum of Ochraceopyronide (**1**) recorded in (DMSO-d_6) at 400 MHz at 60 °C......... 19

Figure S12: ^{1}H NMR spectrum of Ochraceopyronide (**1**) recorded in (DMSO-d_6) at 400 MHz at 80 °C......... 20

Figure S13: ^{13}C NMR spectrum of Ochraceopyronide (**1**) recorded in (DMSO-d_6) at 100 MHz at 80 °C....... 21

Figure S14: ^{1}H NMR spectrum of Ochraceopyronide (**1**) recorded in (MeOD-d_4) at 600 MHz....................... 22

Figure S15: HMBC spectrum of Ochraceopyronide (**1**) recorded in MeOD-d_4) at 600 MHz............................23

Figure S16: HSQC DEPT spectrum of Ochraceopyronide (**1**) recorded in (MeOD-d_4) at 600 MHz...................24

Figure S17: ^{1}HNMR spectrum of compound **2** recorded in (MeOD--d_4) at 400 MHz.................................... 25

Figure S18: HSQC DEPT spectrum of compound (**2**) recorded in (MeOD--d_4) at 400 MHz............................ 26

Figure S19: HMBC spectrum of compound (**2**) recorded in (MeOD--d_4) at 400 MHz.................................... 27

Figure S20: ^{1}HNMR spectrum of compound (**3**) recorded in CDCl3 at 400 MHz... 28

Figure S21: ^{13}CNMR spectrum of compound (**3**) recorded in CDCl3 at 100 MHz... 29

Figure S22: ^{1}HNMR spectrum of compound (**4**) recorded in MeOD-d_4 at 400 MHz....................................... 30

Figure S23: ^{13}C NMR spectrum of compound **4** recorded in MeOD-d_4 at 400 MHz....................................... 31

Figure S24: ^{1}HNMR spectrum of compound (**5**) recorded in DMSO-d_6 at 400 MHz.. 32

Figure S25: HSQC DEPT spectrum of compound (**5**) recorded in DMSO-d_6 at 400 MHz................................. 33

Figure S26: HMBC spectrum of compound (**5**) recorded in DMSO-d_6 at 400 MHz……………………………………….34

Figure S27: Calculated torsion angles *Φ* and ^3J (Hz) for H1-H2, H2-H3 and H3-H4 of the E and T conformers of 1……35

Figure S28: Theoretical $^3J_{HH}$ coupling constants for each of the three pairs H1-H2, H2-H3, H3-H4, for the dihedral angle range -60 to 60, based on the Altona equation………………………………………………………………………….36

Figure S29: Distribution of furanose conformers of *β*-D- and *β*-L-C-lyxofuranoside residues of Ochraceopyronide, after Molecular Dynamics simulation……………………………………………………………………………………………..37

Figure S30: Pseudorotation wheel for Ochraceopyronide ………………………………………………………………………38

Table S1: NMR data of Ochraceopyronide (1) in MeOD at 600 MHz, δ ppm[a]

Position	^{1}HNMR (*J* in Hz)	^{13}CNMR	COSY	HMBC
1	-	-	-	-
2	-	166.2	-	-
3	5.64 (d, 8.1)	100.6	4	2, 4
4	7.84 (d, 8.1)	143.9	3	2, 3, 6, 1'
5	-	nd	-	-
6	-	152.0	-	-
1'	6.13 (d, 4.2)	87.1	2'	4, 6. 2'
2'	4.16 (dd, 2.9, 4.2)	76.8	1', 3'	1', 3'
3'	4.07 (br t, 3.2)	77.4	4'	1', 2', 4', 5'
4'	3.92 (ddd, 3.8, 3.9, 5.1)	86.2	3', 5'	3', 5'
5'	a 3.79 (dd, 5.2, 11.8)	62.2	4', 5'b	3'. 4'
	b 3.81 (dd, 3.9, 11.8)		4', 5'a	

[a] The assignments were based on DEPT, ^{1}H-^{1}H COSY and HMBC experiments and recorded in MeOD-d_4.

Table S2: NMR data of isotorachrysone 6-O-α-D-ribofuranoside (2) at 400 MHz[a]

Position	^{1}HNMR (J in Hz)	HSQC	COSY	HMBC
1	-	154.2	-	-
2	-	124.0	-	-
3	-	135.8	-	-
4	7.01 s	120.3	3'	1, 2, 3', 5, 9, 10
5	7.00 d (2.1)	104.6	7	4, 6, 7, 9
6	-	158.5	-	-
7	6.78 d (2.1)	99.8	5	5, 6, 9
8	-	159.2	-	-
9	-	110.0	-	-
10	-	139.2	-	-
1'	-	206.8	-	-
2'	2.59 s	32.3	-	2, 1'
3'	2.29 s	19.9	4	2, 3, 4
4'	4.08 s	56.8	-	8
1"	5.77 d (4.5)	101.9	2"	4", 6
2"	4.24 dd (4.5, 6.5)	73.2	1"	3"
3"	4.13 dd (3.1, 6.5)	71.1	2	1", 2", 5"
4"	4.17 br.q (3.5)	87.7	5"	-
5"	3.72 d (12.1, 3.5)<br>3.69 d (12.1, 4.0)	63.1	4"	-

[a] The assignments were based on DEPT, HSQC, and HMBC experiments recorded in MeOD-d_4.

Table S3: NMR data of 2-OH-diplopterol (3) at 400MHz, δ ppm[a]

Position	^{1}HNMR (J in Hz)	^{13}CNMR	COSY	HMBC	NOESY
1	0.67 dd (2.0, 12.5)	49.7	2, 3	2, 3, 5, 10	11
	2.04 dq (2.0, 11.4)				
2	3.87 tt (4.1,11.4)	65.4	1, 3		24
3	1.06 t (12.0)	51.2	2, 1	1, 2, 4, 23, 24	7, 20
	1.72 m				
4	-	35.0	-	-	-
5	0.68 s	55.5	6	2, 9, 10, 24	
6	1.30 (d, 3.9)	18.4	7	5, 7, 8, 9	24
	1.50 m				
7	1.22 m	33.1	6	5, 8	
	1.36 d (3.2)				
8	-	41.9	-	-	-
9	1.28 br.t (4.3)	50.3	11	10, 11, 14, 27	
10	-	39.2	-	-	-
11	1.31 d (3.8)	21.0	9, 12	7, 9, 12	1, 26
	1.54 d (4.3)				
12	1.41 br.d (3.8)	24.0	11, 13	11, 13, 14, 28	
	1.48 br.t (4.3)				
13	1.34 br.d (3.8)	49.8	12	7, 9, 12, 14	
14	-	41.9	-	-	-
15	1.20 m	34.4	16	14, 27	
	1.44 m				
16	1.56 dd (3.6, 12.9)	21.9	15, 17	15, 17, 18	
	1.91 dt (2.4, 12.9)				
17	1.43 br.t (3.6)	53.9	16, 21	14, 16, 21, 28	
18	-	44.1	-	-	-
19	0.92 m	41.2	20	17, 20, 21, 28	
	1.51 m				
20	1.47 m	26.6	19, 21	18, 21	
	1.73 m				
21	2.20 br.q (9.0)	51.1	17, 20, 22	17, 18, 20, 22, 29, 30	17, 29
22	-	73.9	-	-	-
23	0.90, 3H, s	33.5	-	3, 4, 5, 24	19
24	0.82, 3H, s	22.4	-	3, 4, 5, 23	6
25	0.84, 3H, s	17.0	-	1, 5, 9, 10	
26	0.93, 3H, s	16.9	-	13, 14	11
27	0.93, 3H, s	16.8	-	13, 14	
28	0.74, 3H, s	16.1	-	13, 17, 18, 19	
29	1.16, 3H, s	28.7	-	21, 22, 30	
30	1.19, 3H, s	30.9	-	21, 22, 29	

[a] The assignments were based on DEPT, ^{1}H-^{1}H COSY, HSQC, and HMBC experiments and recorded in $CDCl_3$.

Table S4 : NMR data of questin (4) at 400 MHz, δ ppm[a]

Position	^{1}HNMR (J in Hz)[b]	^{1}HNMR (J in Hz)	^{13}CNMR	COSY	HMBC
1	-	-	163.7	-	-
2	7.11 br.d (1.7)	7.08 s	125.3	4, 3Me	4, 9a, 3Me
3	-	-	148.3	-	-
4	7.50 br.d (1.7)	7.51 s	120.6	2, 3Me	2, 9a, 10, 3Me
4a	-	-	133.9	-	-
5	7.35 d (2.3)	7.28 d (2.3)	108.4	7	7, 8a, 10
6	-	-	165.2	-	-
7	6.95 d (2.3)	6.83 d (2.3)	105.7	5, 8-OMe	5, 8, 8a
8	-	-	166.2	-	-
8a	-	-	114.5	-	-
9-CO	-	-	184.0	-	-
9a	-	-	116.0	-	-
10-CO	-	-	184.0	-	-
10a	-	-	138.8	-	-
1-OH	11.33 s	-	-	-	-
3-CH_3	2.44 s	2.40 s	21.9	2, 4	2, 3, 4
8-OCH_3	3.99 s	3.97 s	56.8	7	8

[a] The assignments were based on DEPT, ^{1}H-^{1}H COSY, HSQC and HMBC experiments and recorded in MeOD-d_4,
[b] Recorded in acetone-d_6.

Table S5 : NMR data of physcion (5) at 600 MHz, δ ppm[a]

Position	^{1}HNMR (J in Hz)[b]	^{1}HNMR (J in Hz)	^{13}CNMR	COSY	HMBC
1	-	-	161.6	-	-
2	7.06 br.s	7.22 br.d (1.4)	124.2	4, 3-Me	1, 4, 9a, 3-Me
3	-	-	148.5	-	-
4	7.60 br.s	7.55 br.d (1.4)	120.5	2, 3-Me	2, 9a, 10, 3-Me
4a	-	-	nd	-	-
5	7.34 d (2.5)	7.21 d (2.5)	107.5	7	4, 6, 8a, 10
6	-	-	166.2	-	-
7	6.67 d (2.5)	6.89 d (2.5)	106.5	5	5, 8, 8a
8	-	-	165.3	-	-
8a	-	-	110.1	-	-
9-CO	-	-	181.6	-	-
9a	-	-	113.5	-	-
10-CO	-	-	181.6	-	-
10a	-	-	nd	-	-
1-OH	12.10 s	12.10 s	-	-	-
3-CH3	2.43 s	2.43 s	21.4	4	2, 3, 4
6-OCH3	3.91 s	3.94 s	56.2	-	6
8-OH	12.30 s	12.30 s	-	-	-

[a] The assignments were based on DEPT, ^{1}H-^{1}H COSY, HSQC, and HMBC experiments and recorded in DMSO-d_6.
[b] Recorded in $CDCl_3$.

Table S6 : Antimicrobial activities of the crude fungal extract in agar diffusion assays (mm diameter).

	PA[a]	BS[b]	SA[c]	CA[d]	AN[e]
Aspergillus sp. MN611443	12	11	16	10	30

[a]*Pseudomonas aeruginosa* ATCC 27853, [b]*Bacillus subtilis* ATCC 6633, [c] *Staphylococcus aureus* ATCC 6538, [d] *Candida albicans* ATCC 10231, [e] *Aspergillus niger* NRRL A-326.

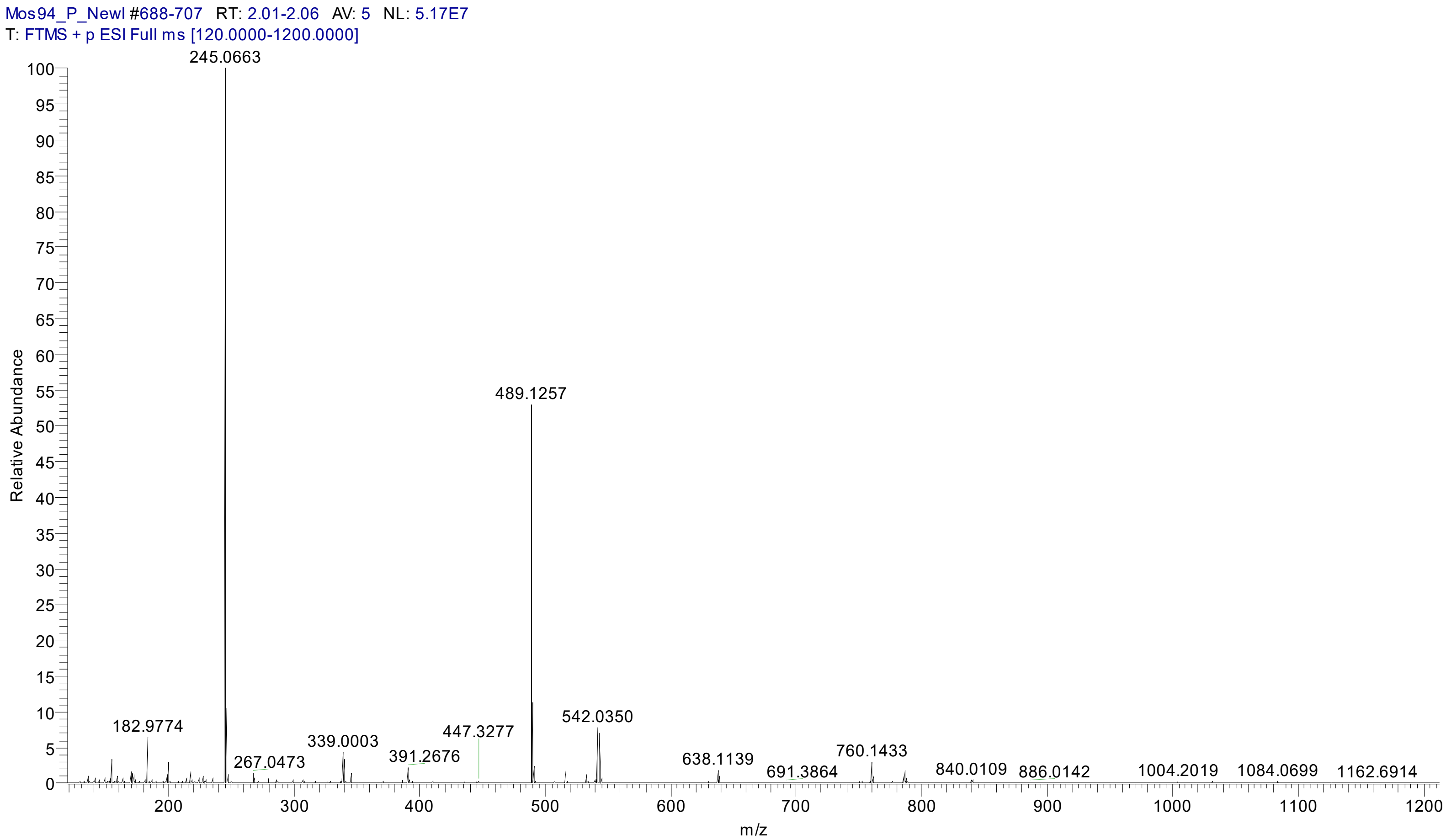

Figure S1: HR-ESI-MS spectrum of compound **1**.

Date	mon aug 03 12:56:43 2020			***Technique***	Infrared
Instrument	Spectrum One	***Spectral Region***	IR	***X Axis***	Wavenumber (cm-1)
Y Axis	%Transmittance	***Spectrum Range***	450.0000 - 4000.0000		
Points Count	3551	***Data Spacing***	1.0000		

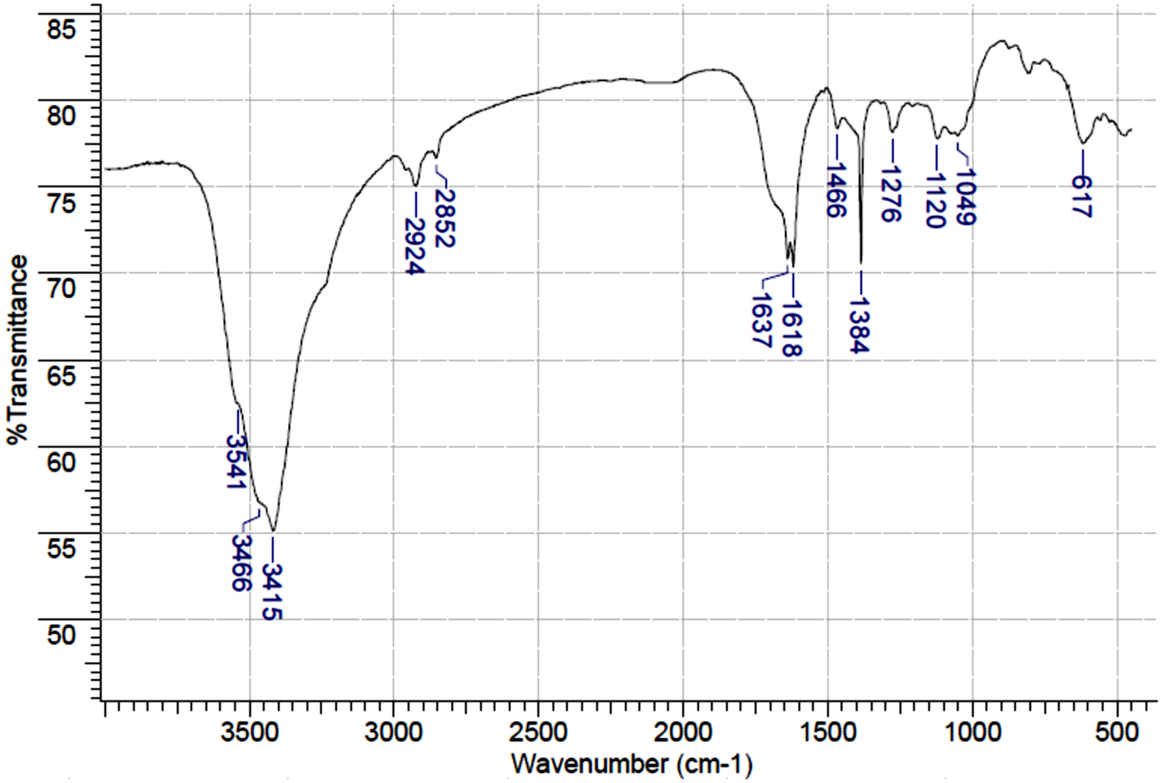

Figure S2: IR spectrum of compound **1** (KBr disc).

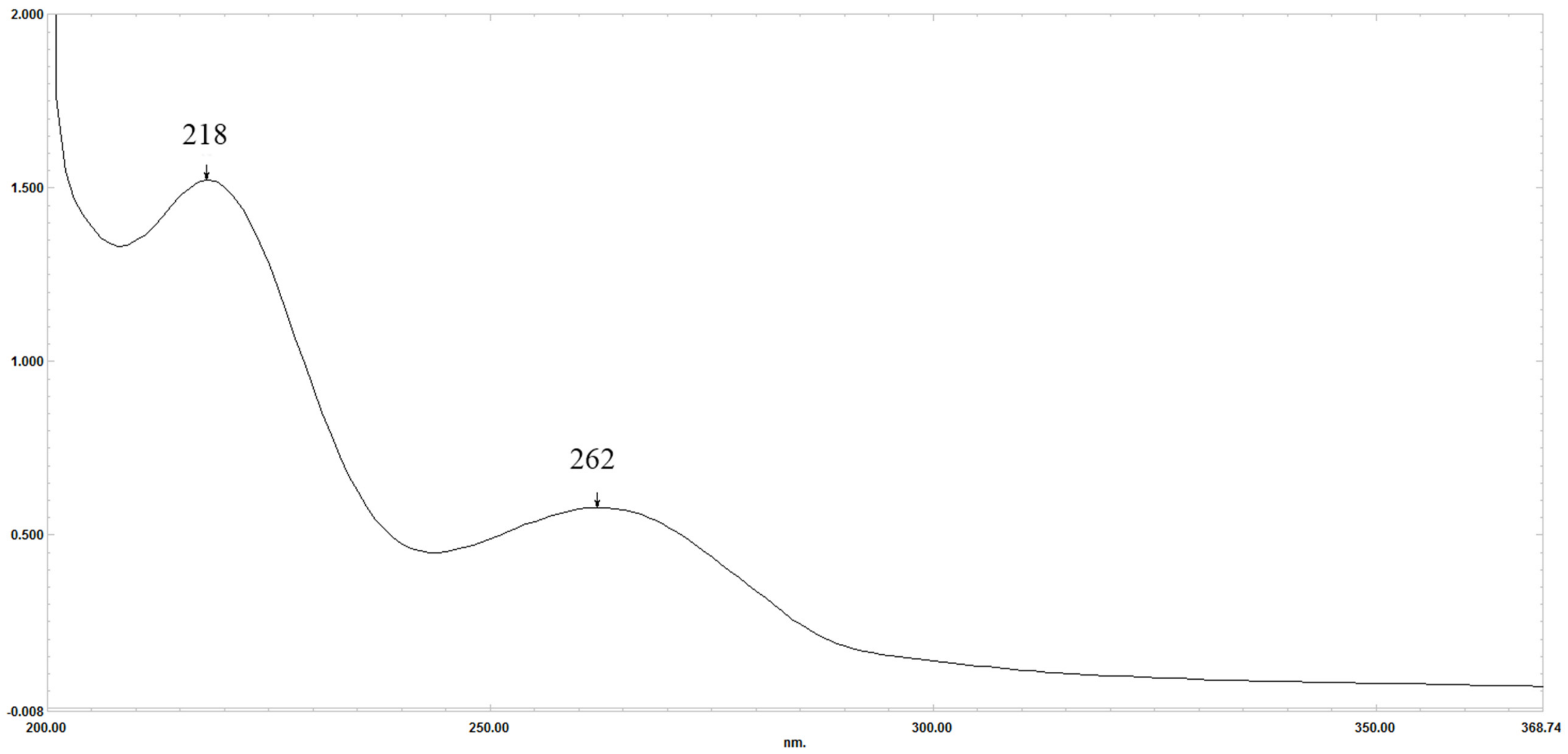

Figure S3 : UV spectrum of **1** in MeOH.

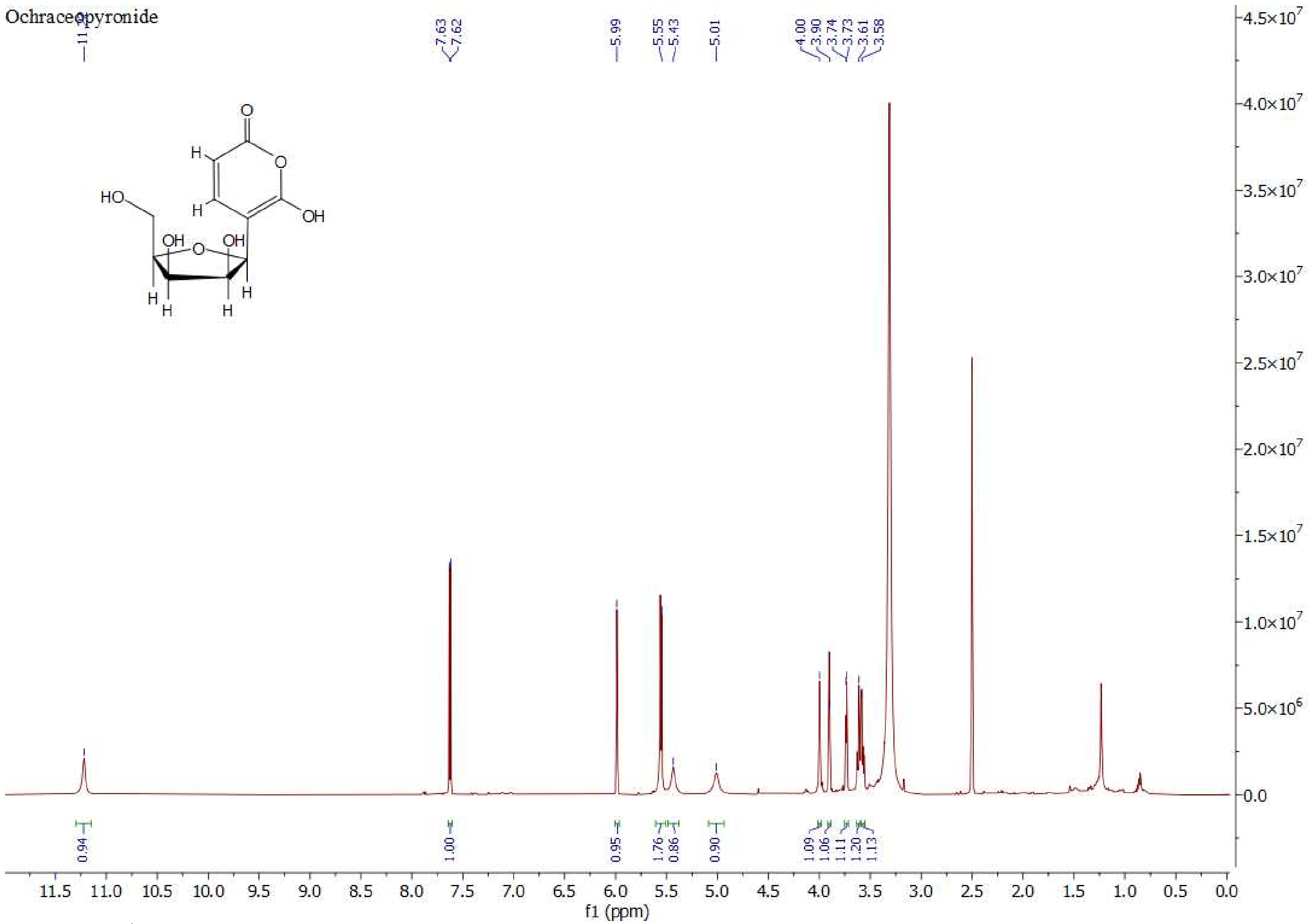

Figure S4: ^{1}H NMR spectrum of compound **1** recorded in DMSO-d_6 at 600 MHz at 298K.

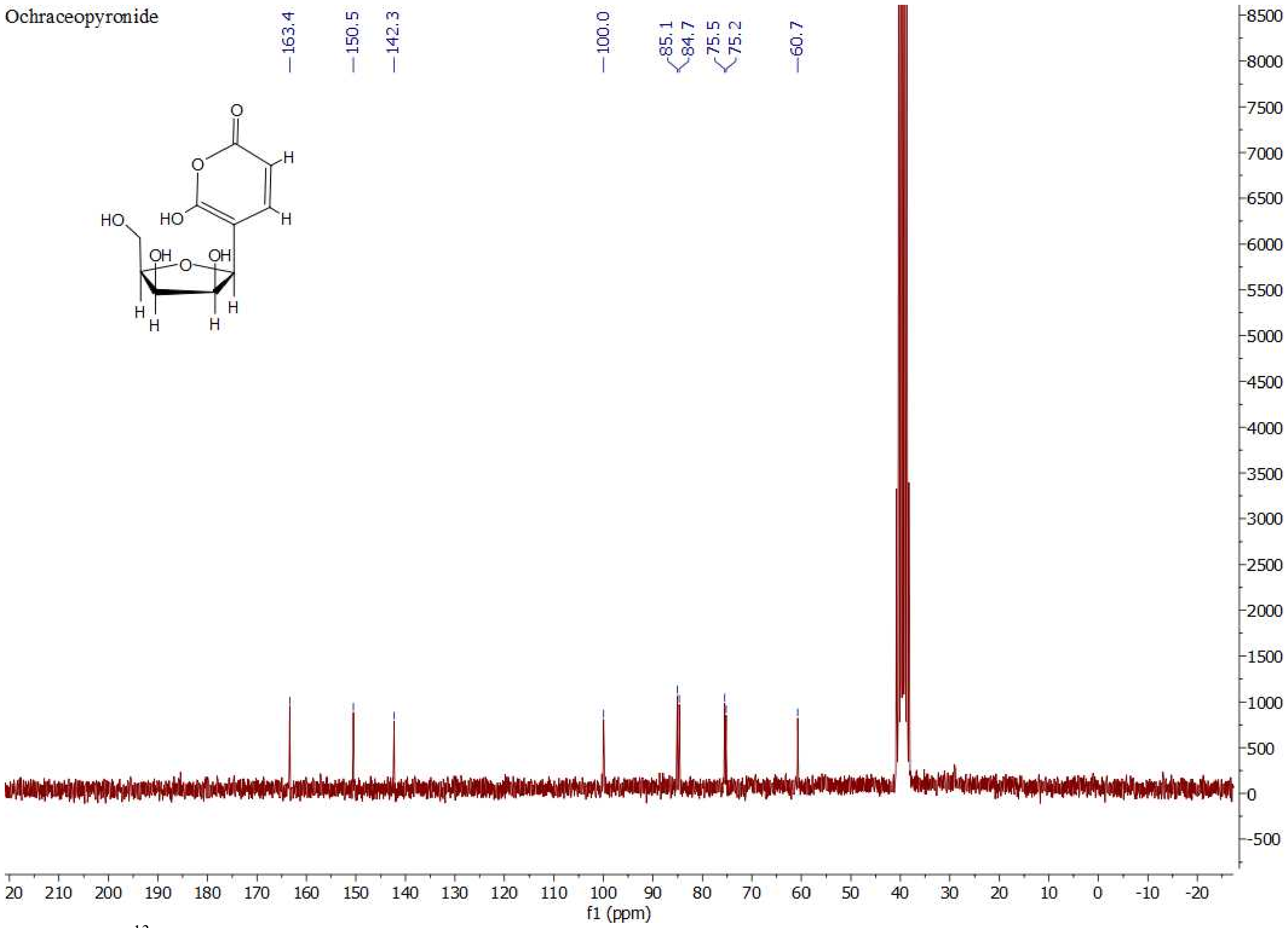

Figure S5 : ^{13}C NMR spectrum of compound **1** recorded in DMSO-d_6 at 150 MHz at 298K

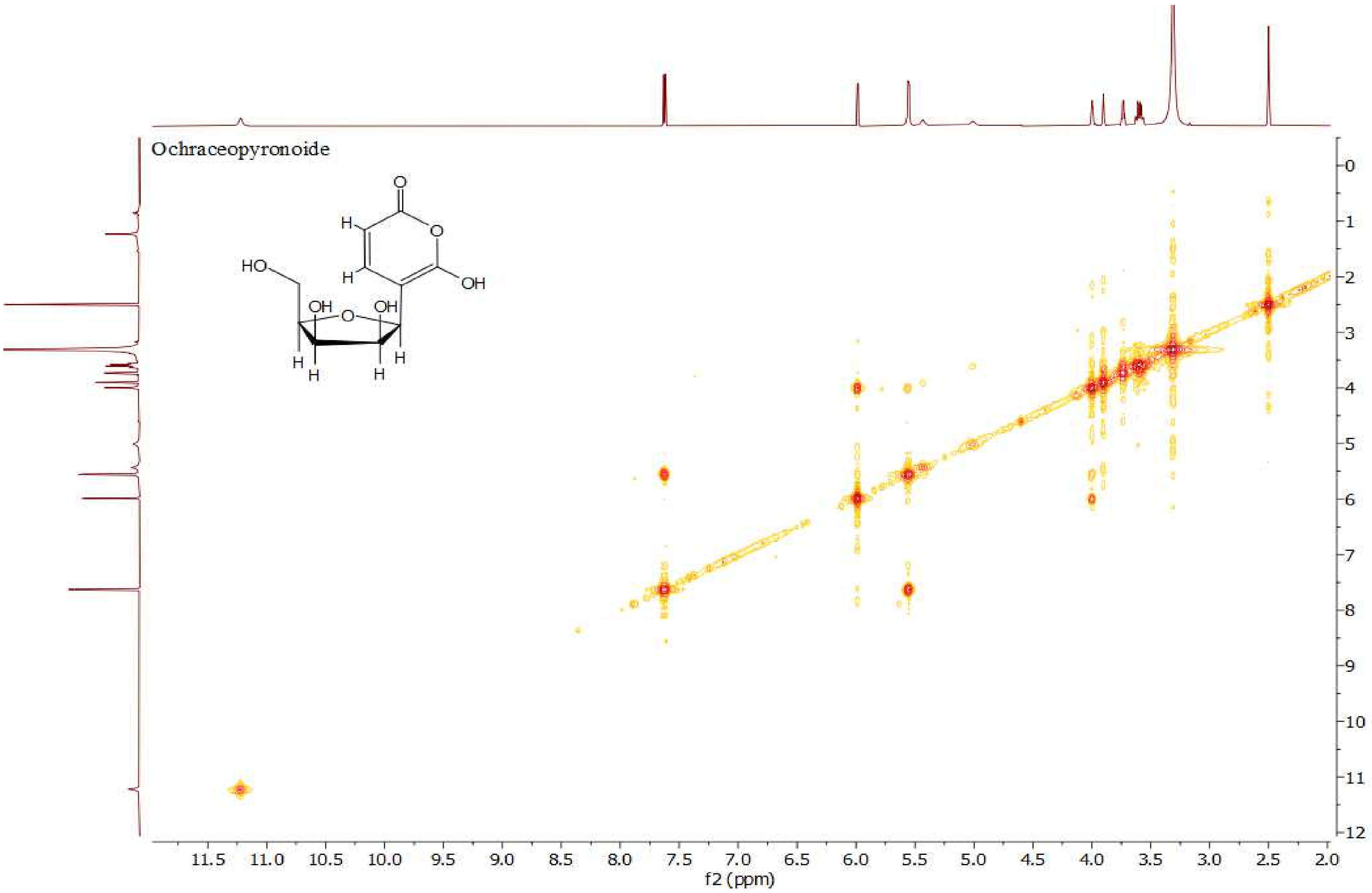

Figure S6 : ^{1}H-^{1}H COSY spectrum of compound **1** recorded in DMSO-d_6 at 600 MHz.

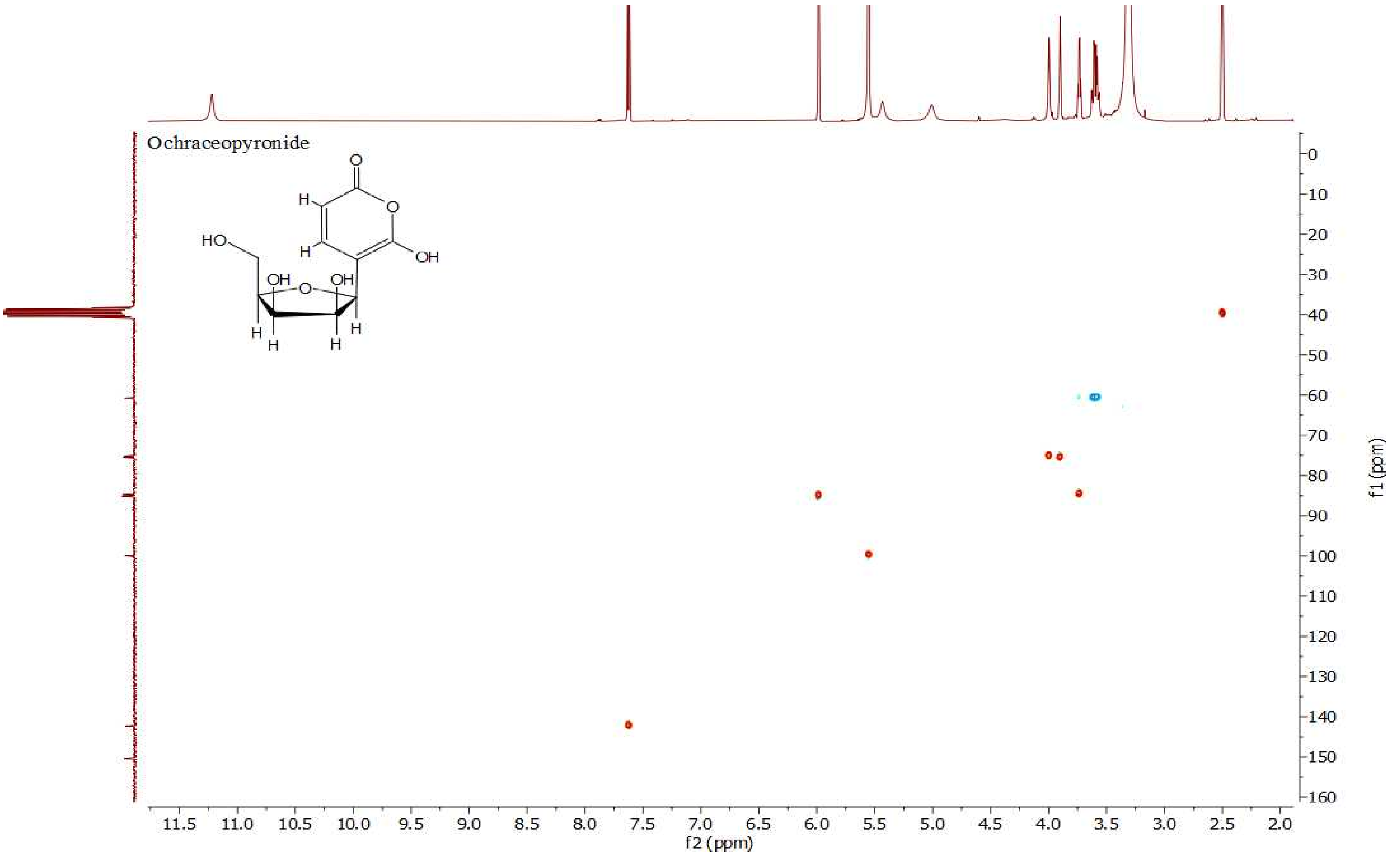

Figure S7: HSQC-DEPT spectrum of compound **1** recorded in DMSO-d_6 at 600 MHz

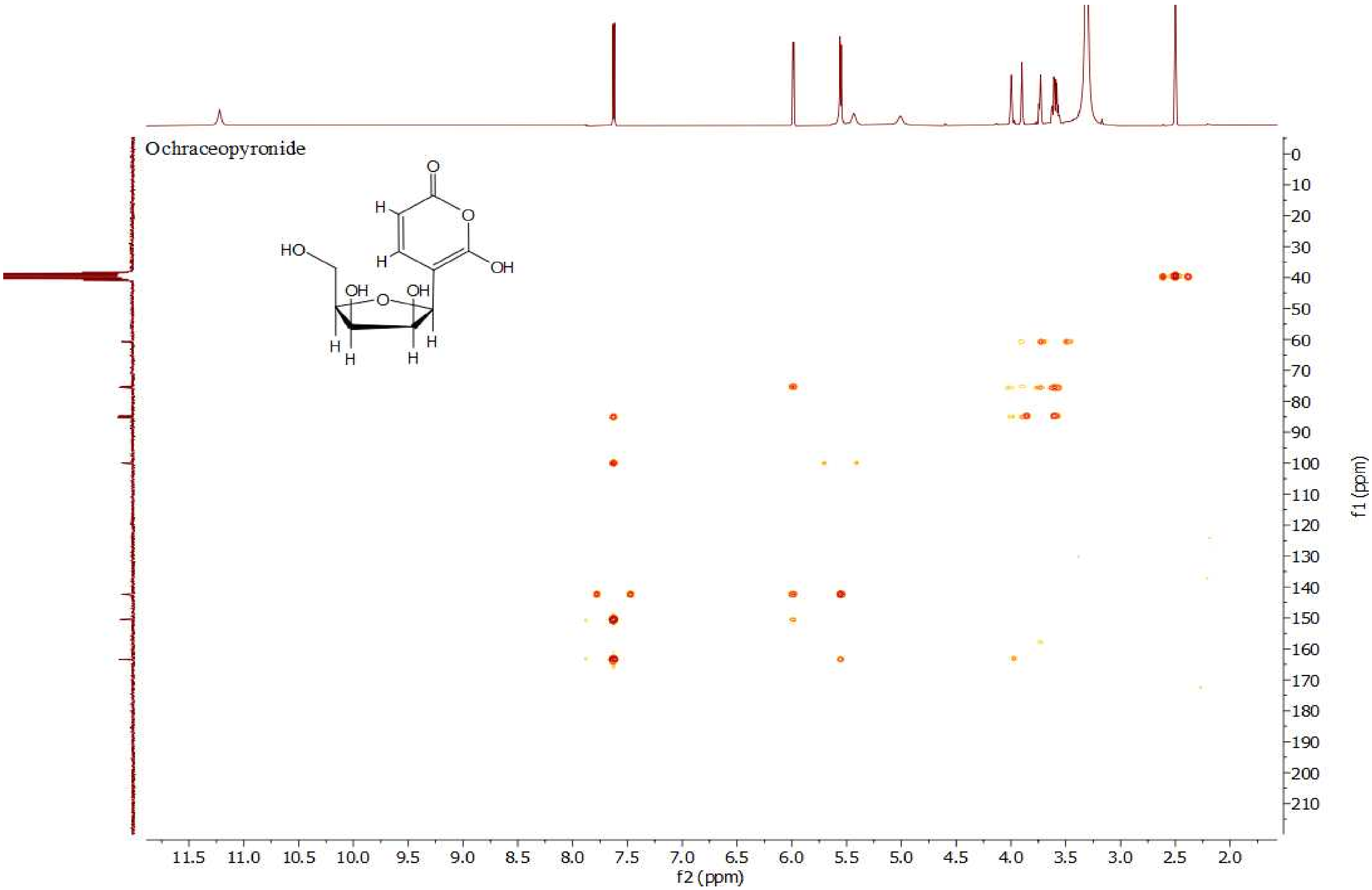

Figure S8: HMBC spectrum of compound **1** recorded in DMSO-d_6 at 600 MHz.

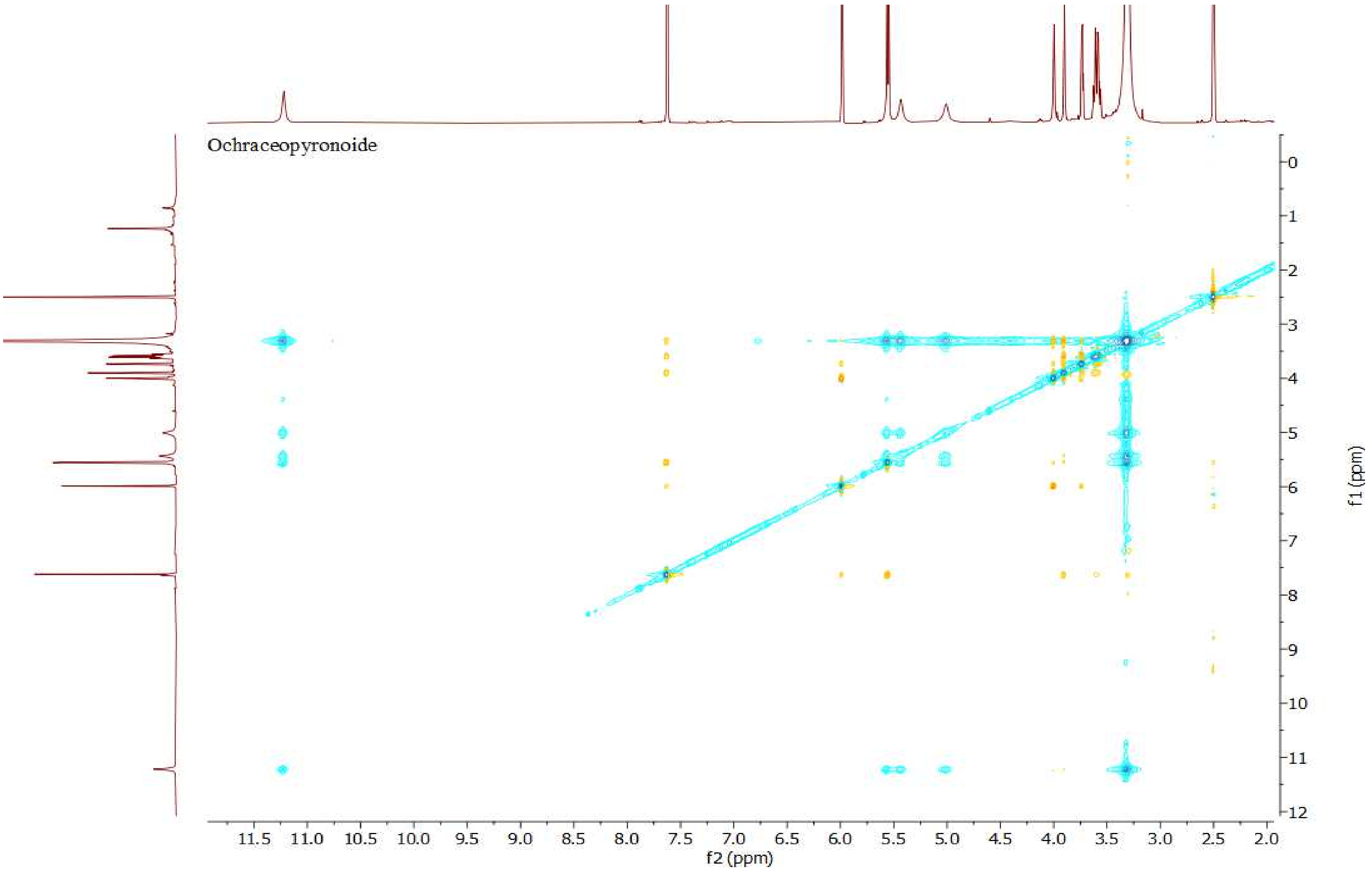

Figure S9: NOESY spectrum of compound **1** recorded in DMSO-d_6 at 600 MHz

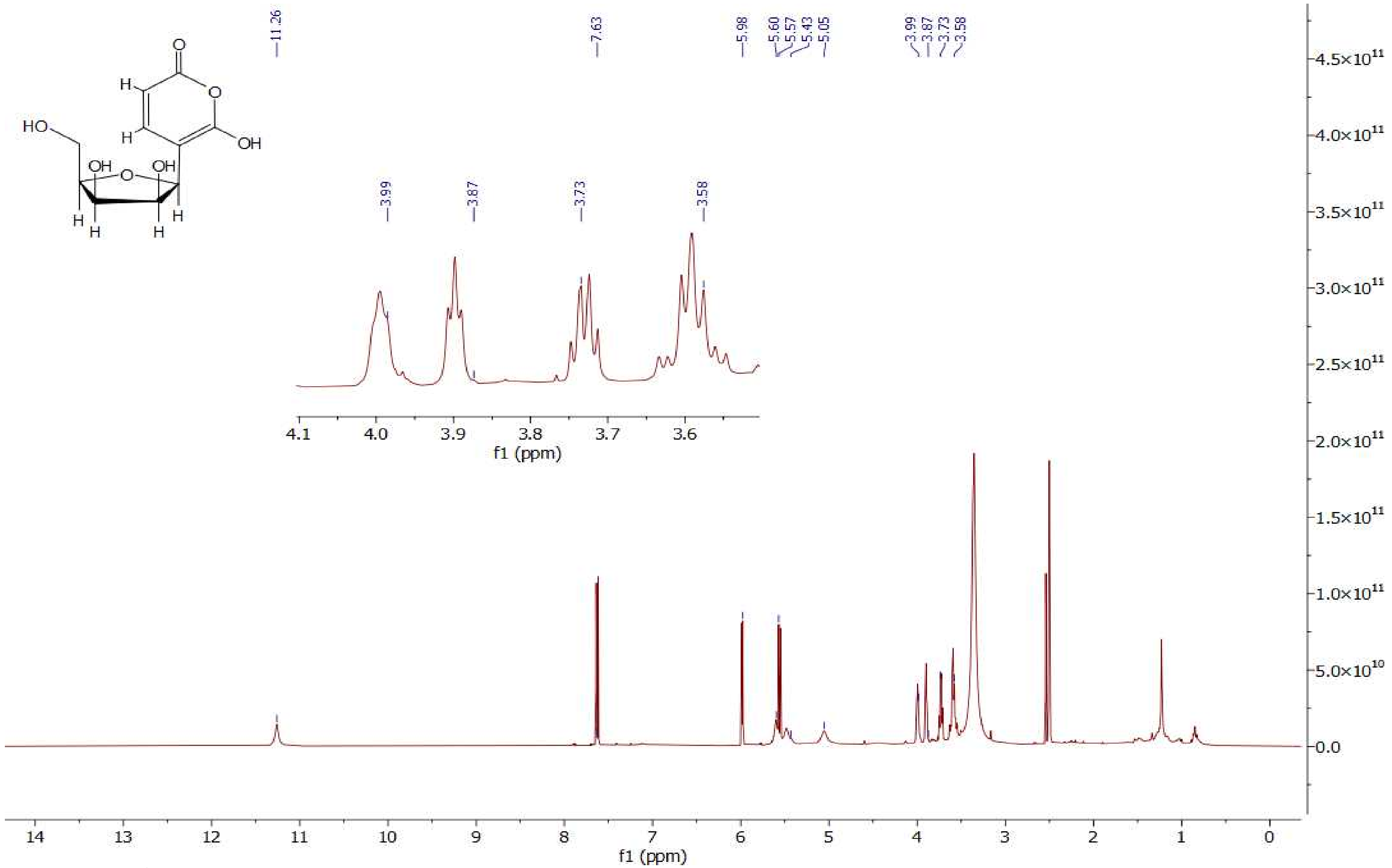

Figure S10 : ^{1}H NMR spectrum of compound **1** recorded in DMSO-d_6 at 400 MHz at 40 °C.

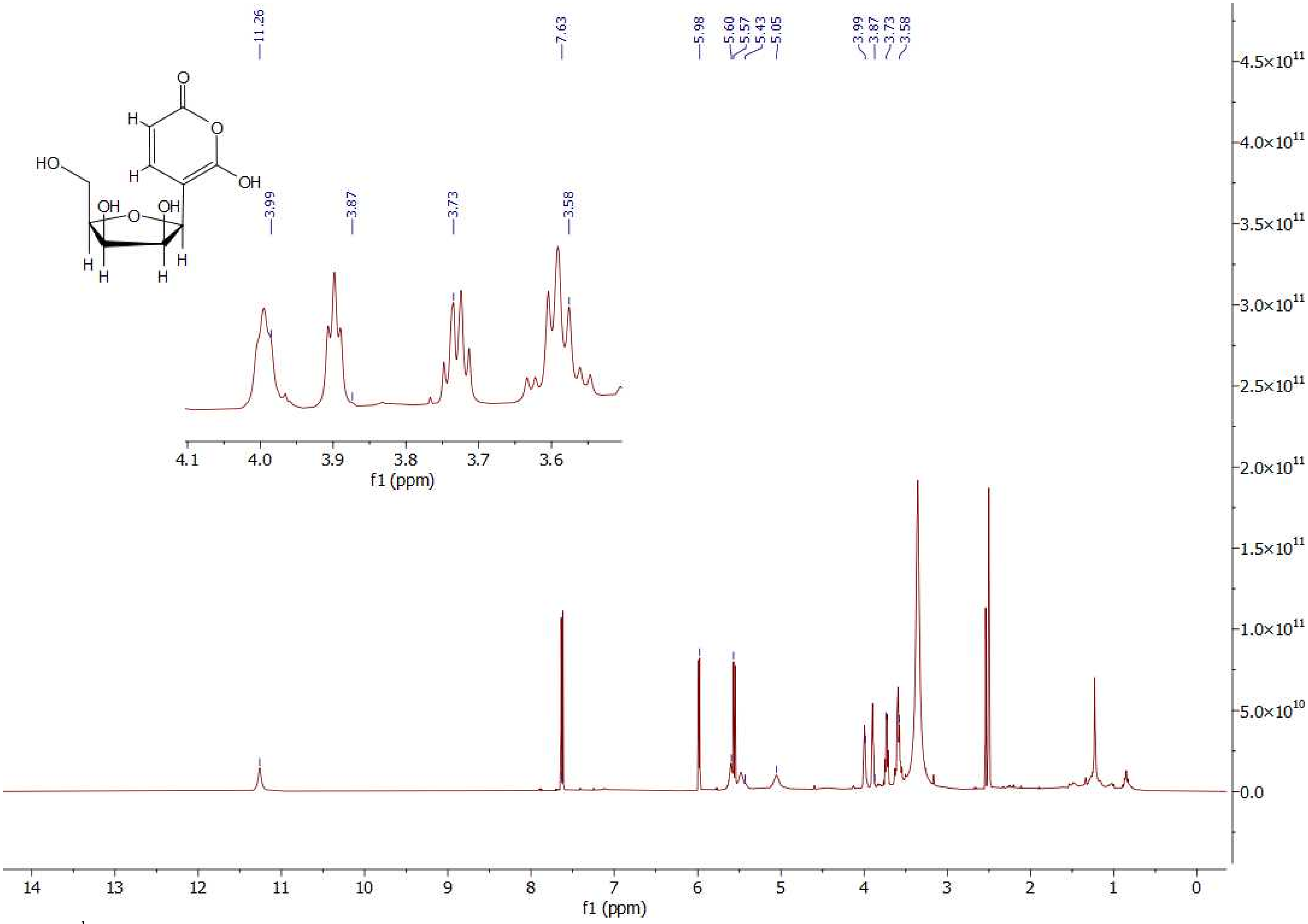

Figure S11: ^{1}H NMR spectrum of compound **1** recorded in DMSO-d_6 at 400 MHz at 60 °C

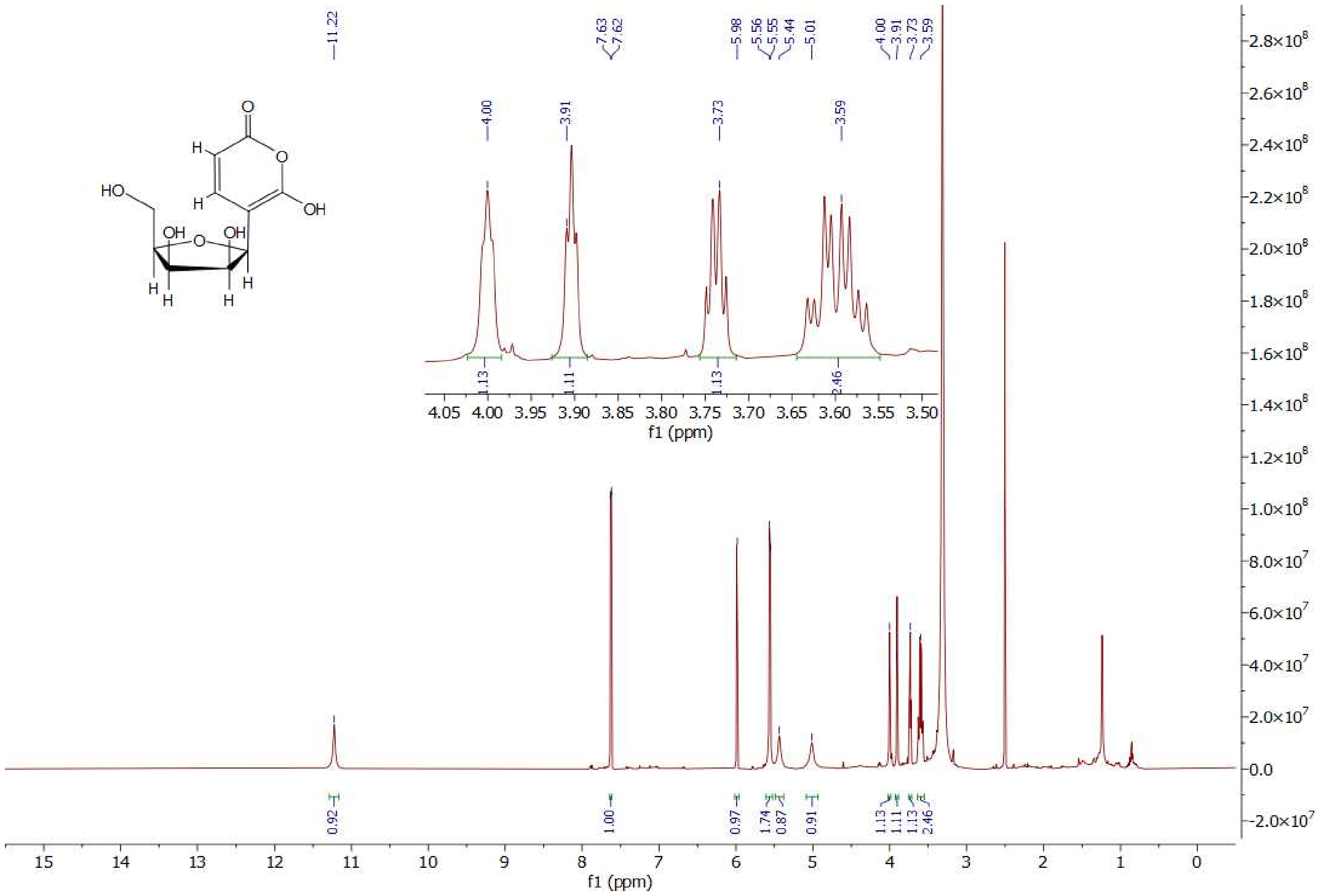

Figure S12 : ^{1}H NMR spectrum of compound **1** recorded in DMSO-d_6 at 400 MHz at 80 °C

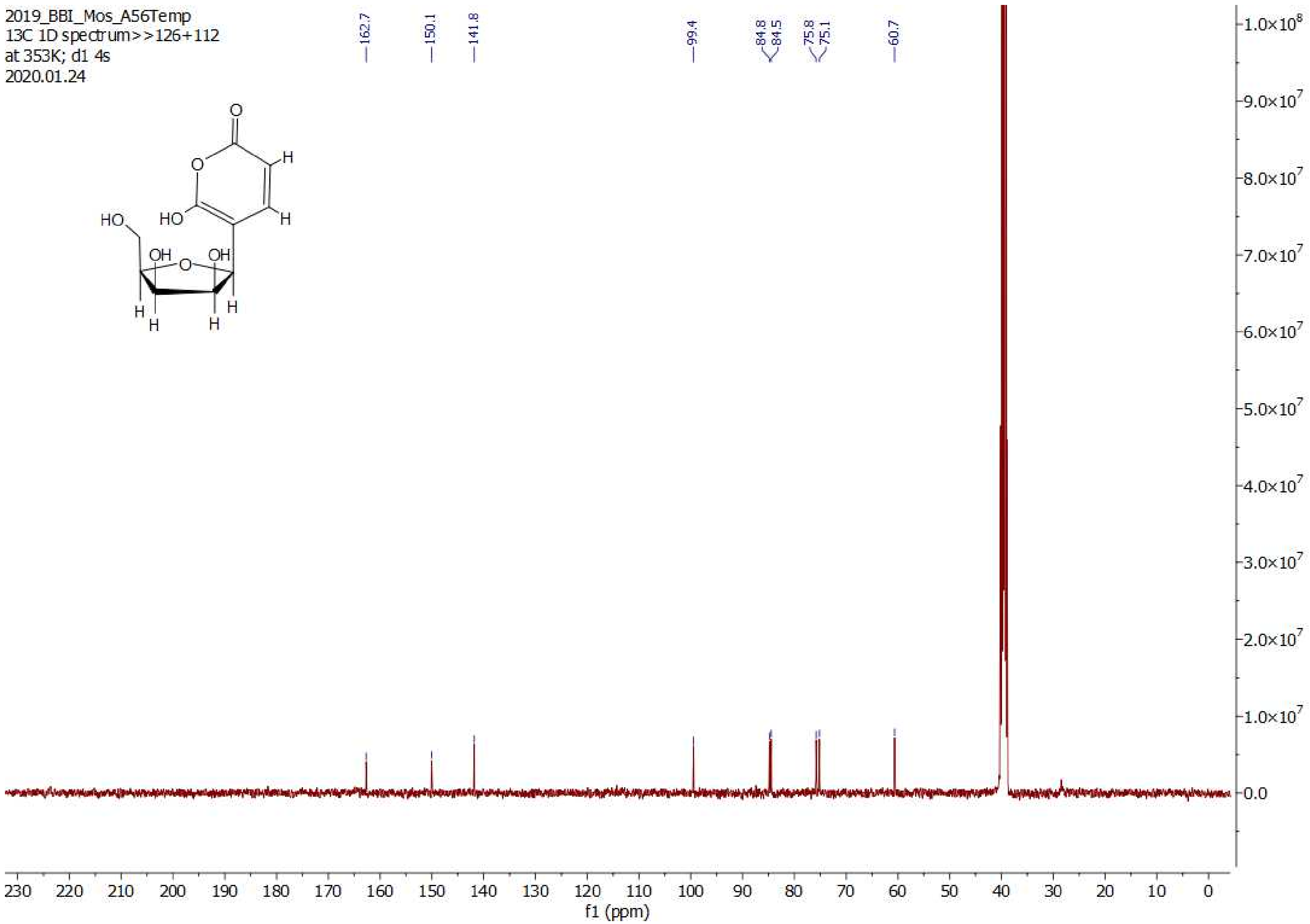

Figure S13 : ^{13}C NMR spectrum of compound **1** recorded in DMSO-d_6 at 100 MHz at 80 °C.

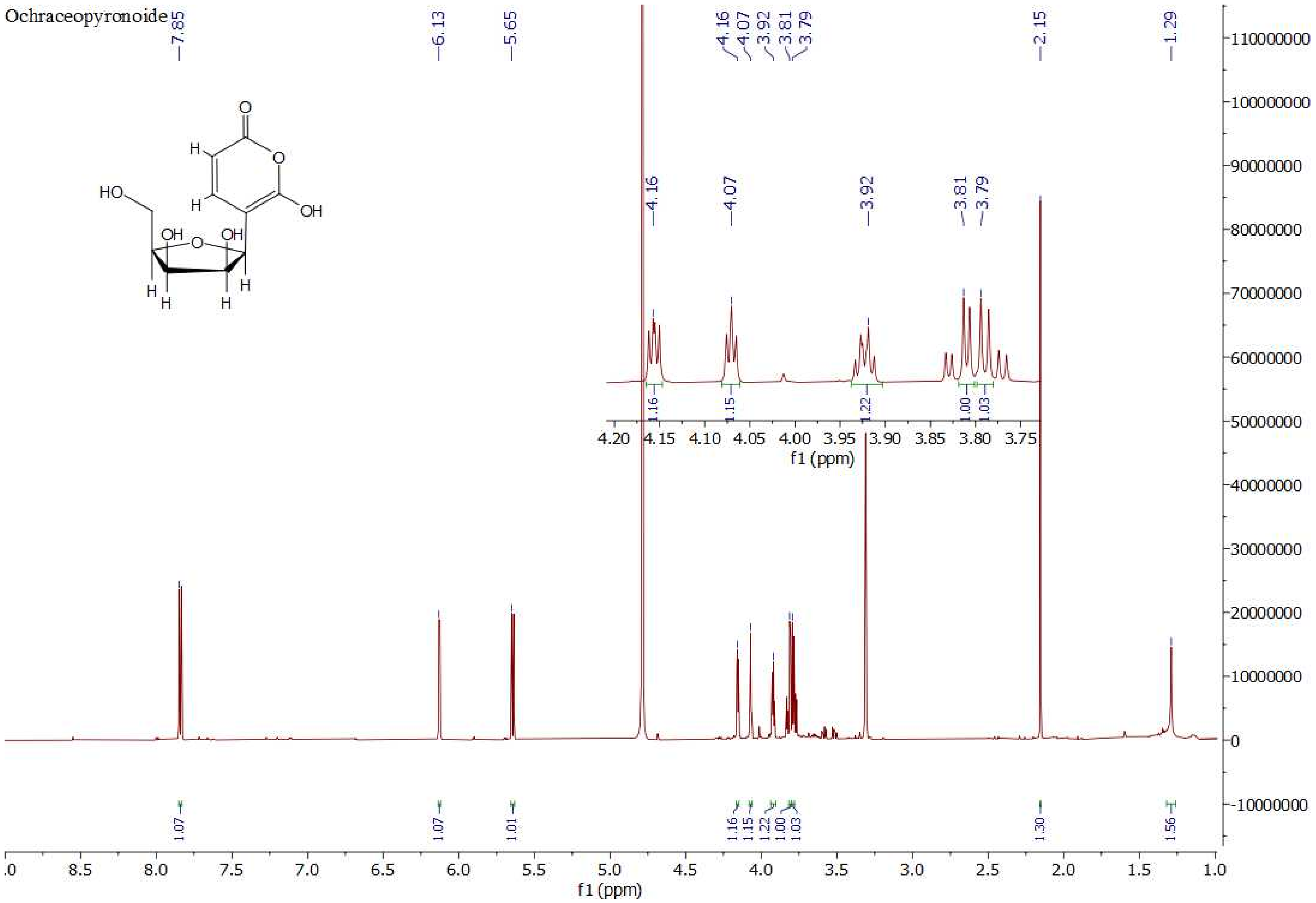

Figure S14: ^{1}H NMR spectrum of compound **1** recorded in MeOD-d_4 at 600 MHz

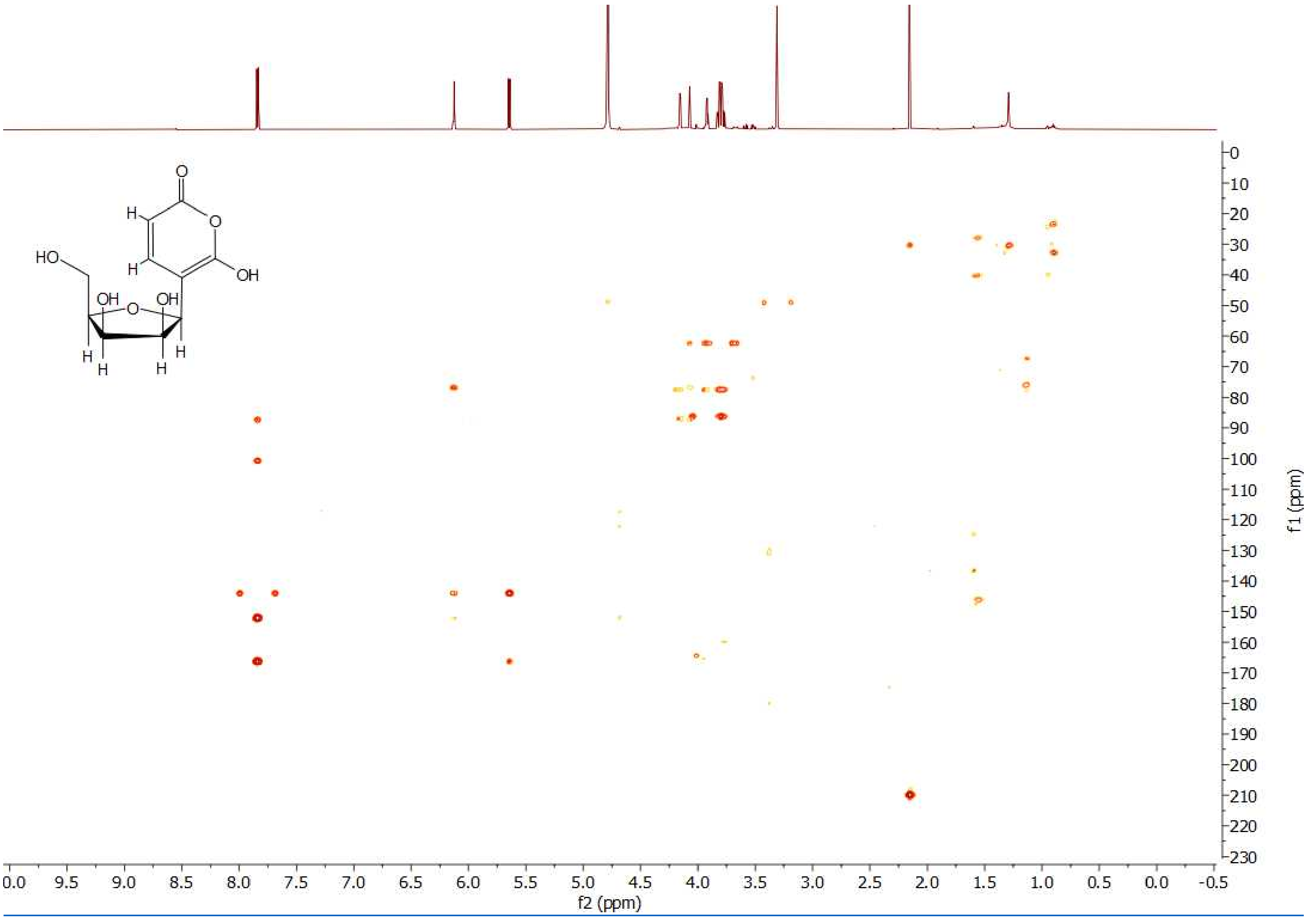

Figure S15: HMBC spectrum of compound 1 recorded in MeOD-d_4 at 600 MHz

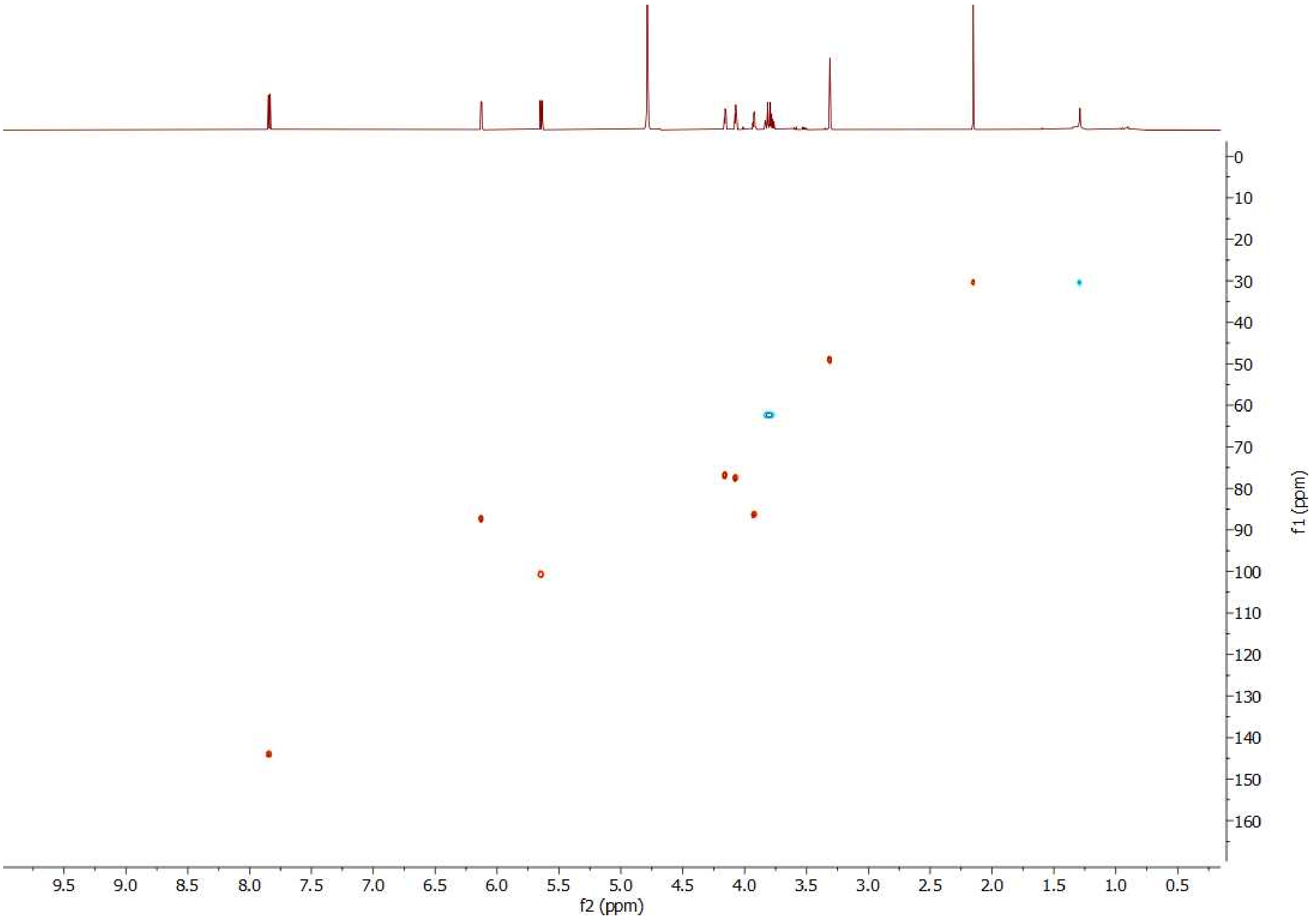

Figure S16: HSQC-DEPT spectrum of compound **1** recorded in MeOD-*d*4 at 600 MHz.

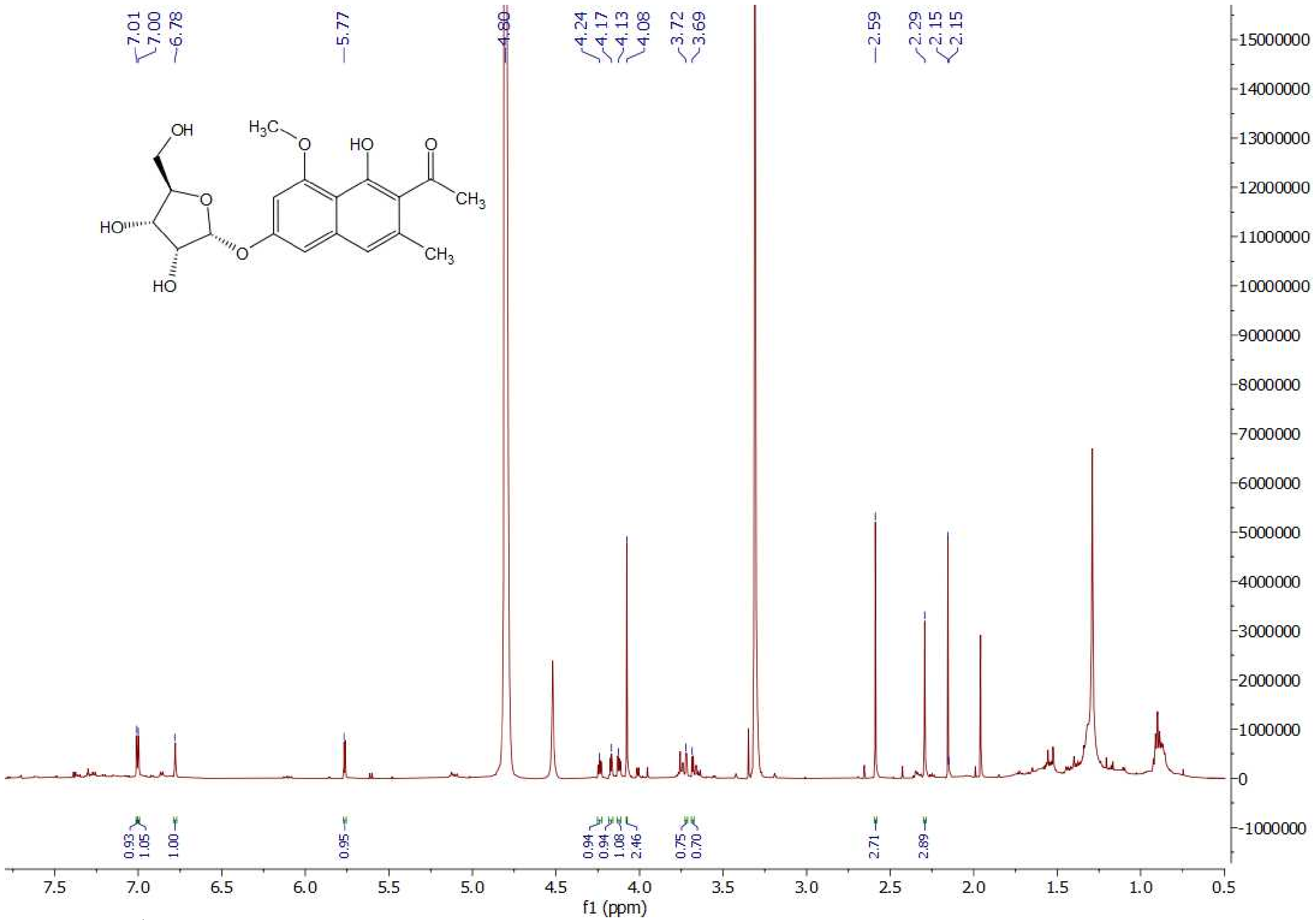

Figure S17: ^{1}HNMR spectrum of compound **2** recorded in MeOD-d_4 at 400 MHz

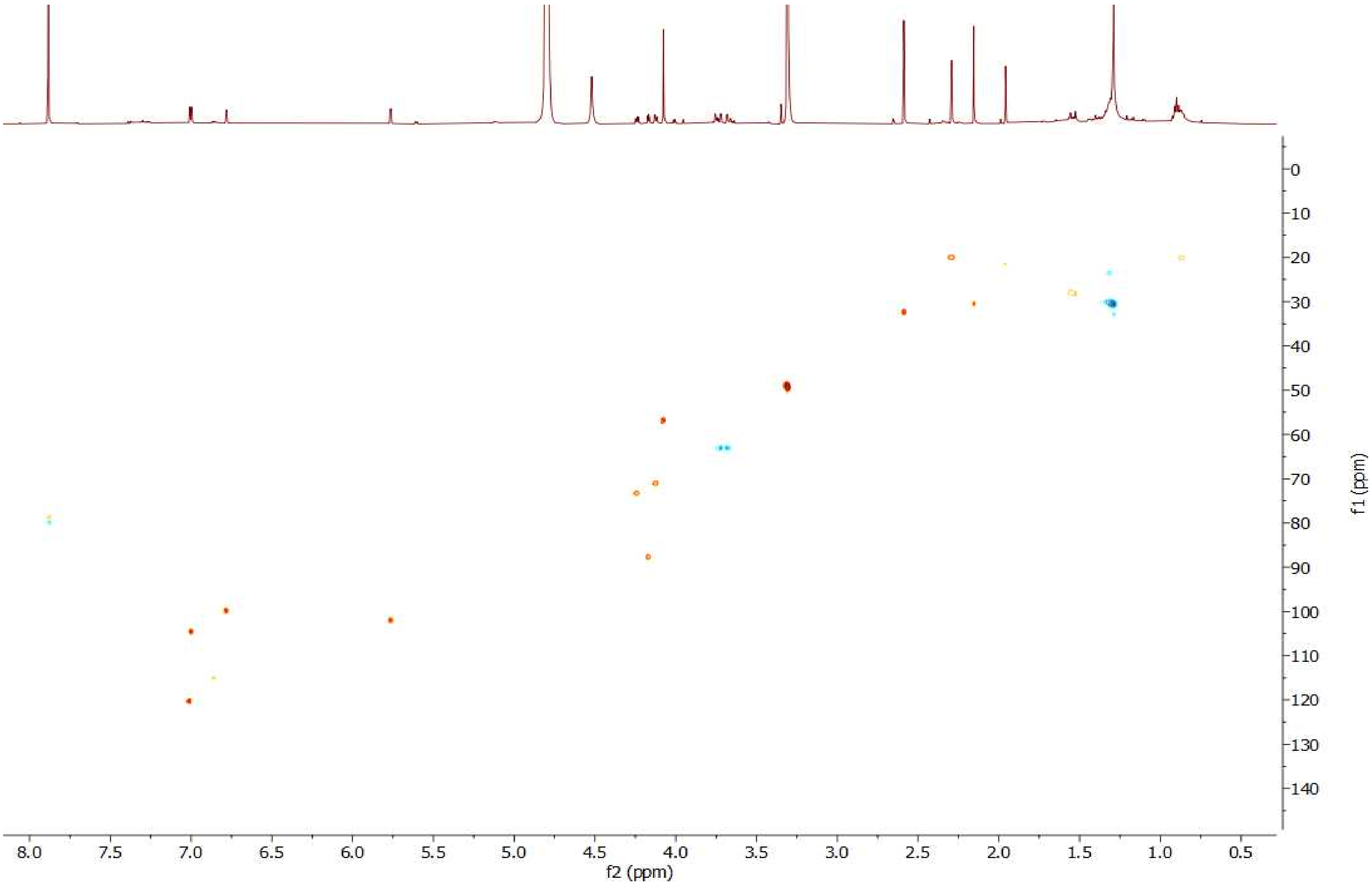

Figure S18: HSQC-DEPT spectrum of compound **2** recorded in MeOD-d_4 at 400 MHz

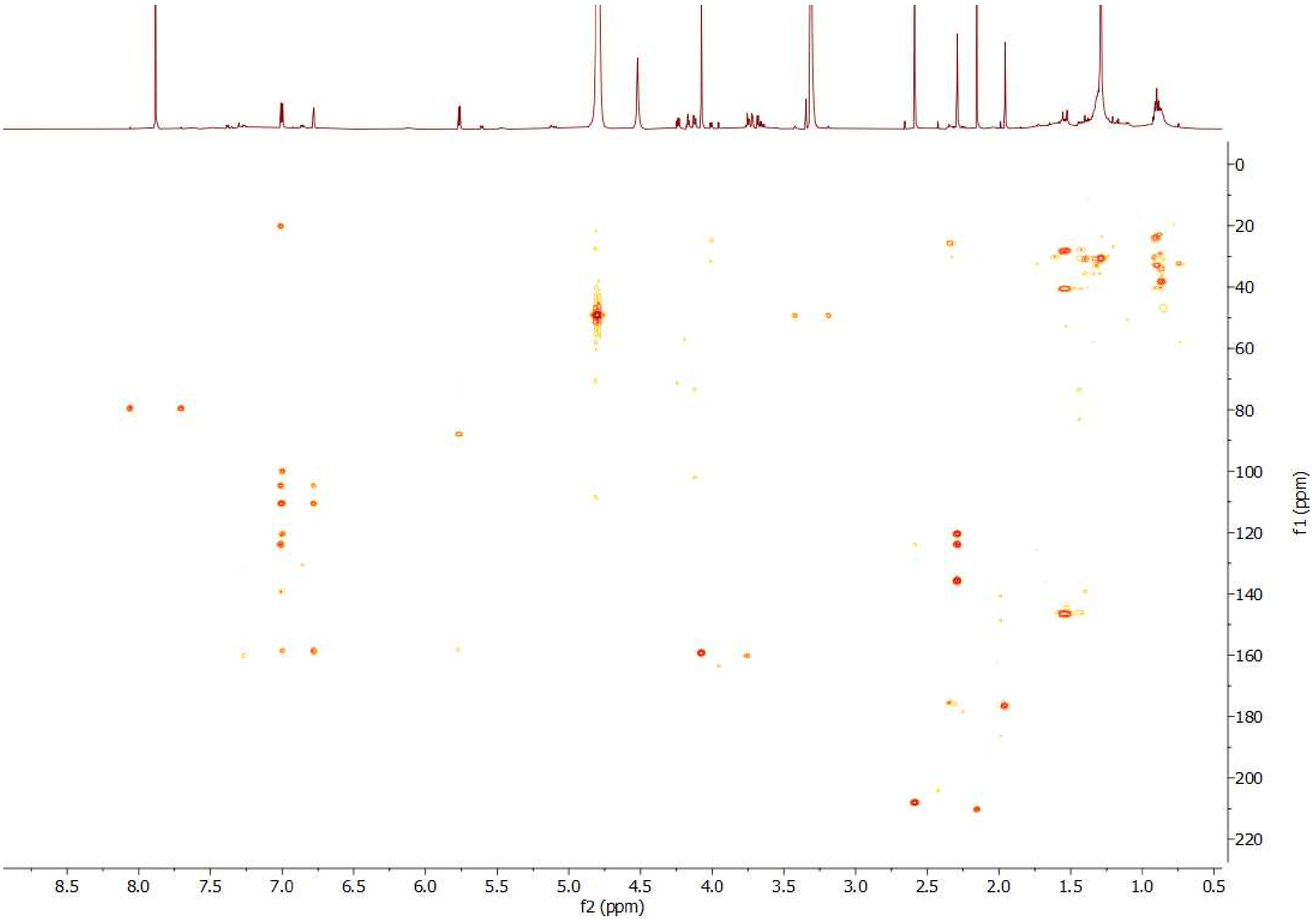

Figure S19: HMBC spectrum of compound **2** recorded in MeOD-d_4 at 400 MHz.

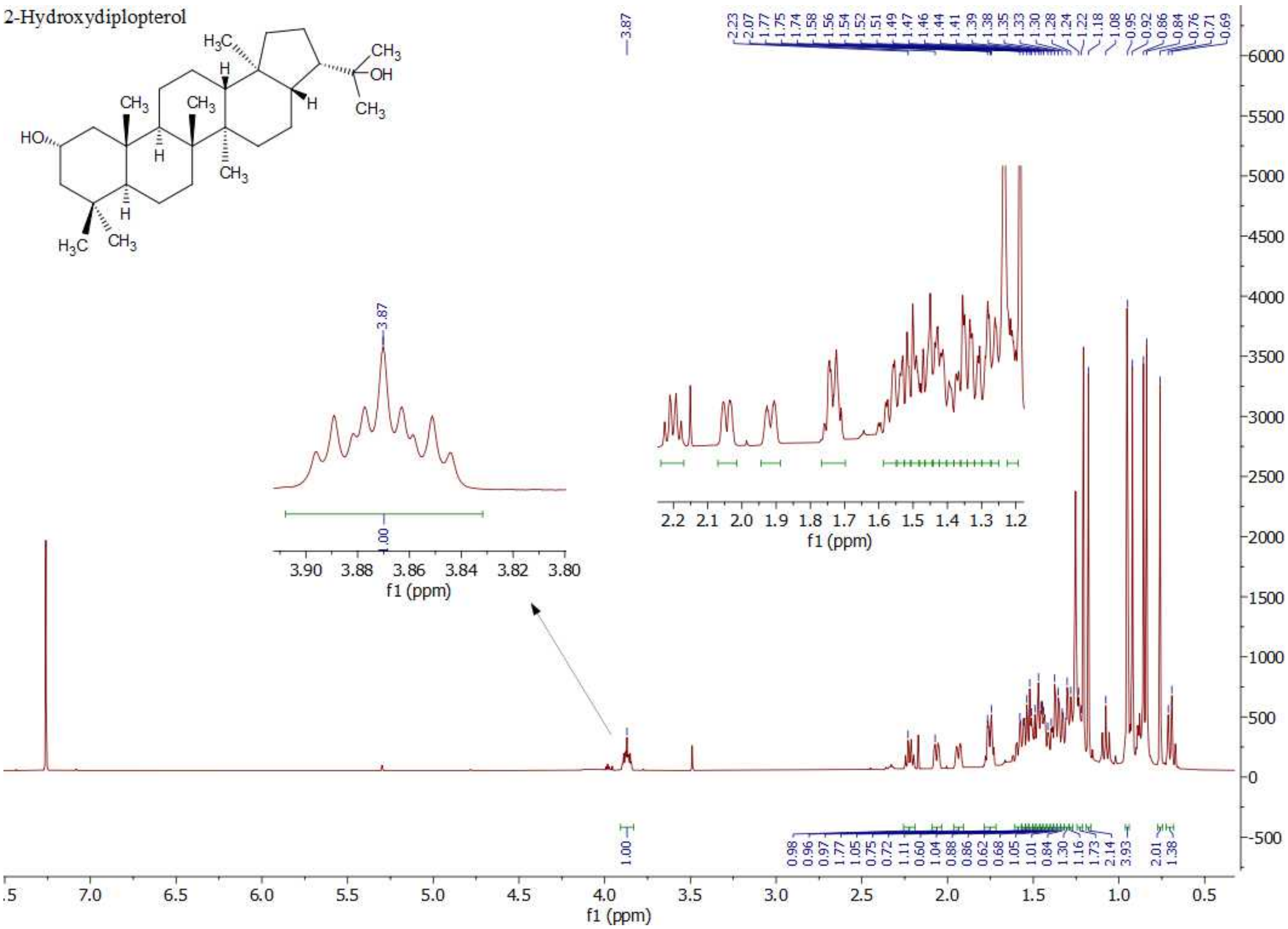

Figure S20 : ^{1}HNMR spectrum of compound **3** recorded in $CDCl_3$ at 400 MHz

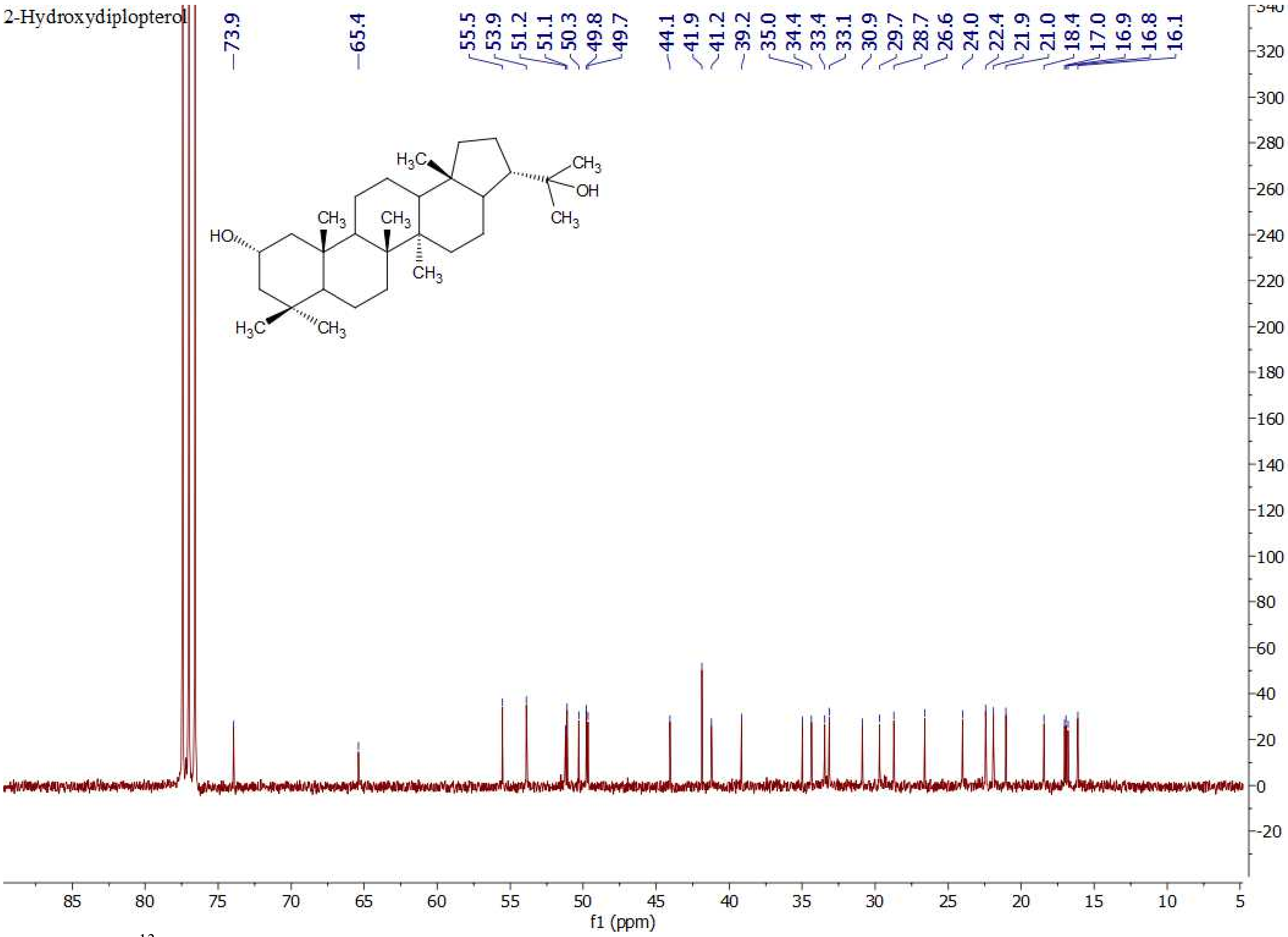

Figure S21: ^{13}CNMR spectrum of compound **3** recorded in CDCl3 at 100 MHz

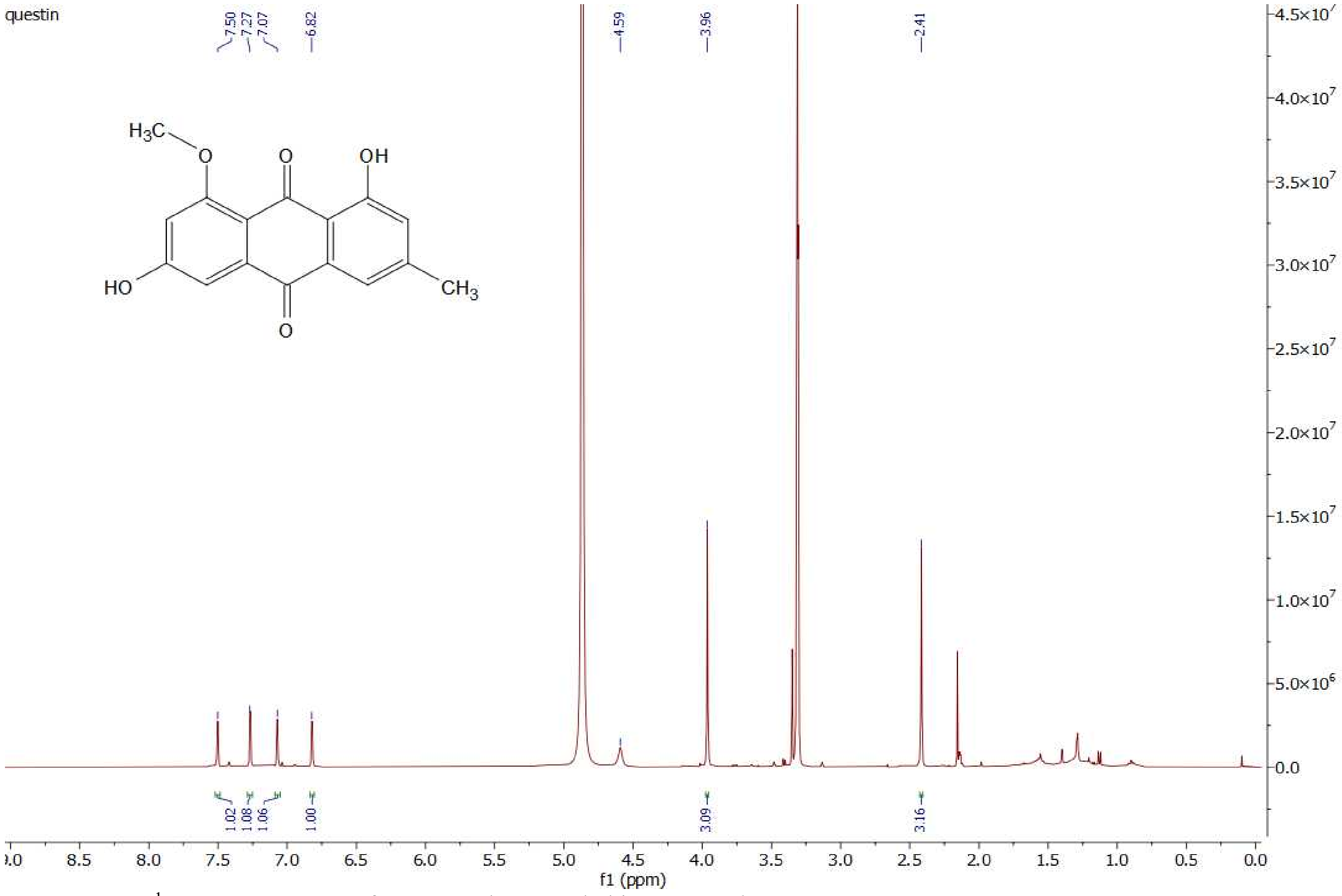

Figure S22: ^{1}HNMR spectrum of compound **4** recorded in MeOD-*d*4 at 400 MHz

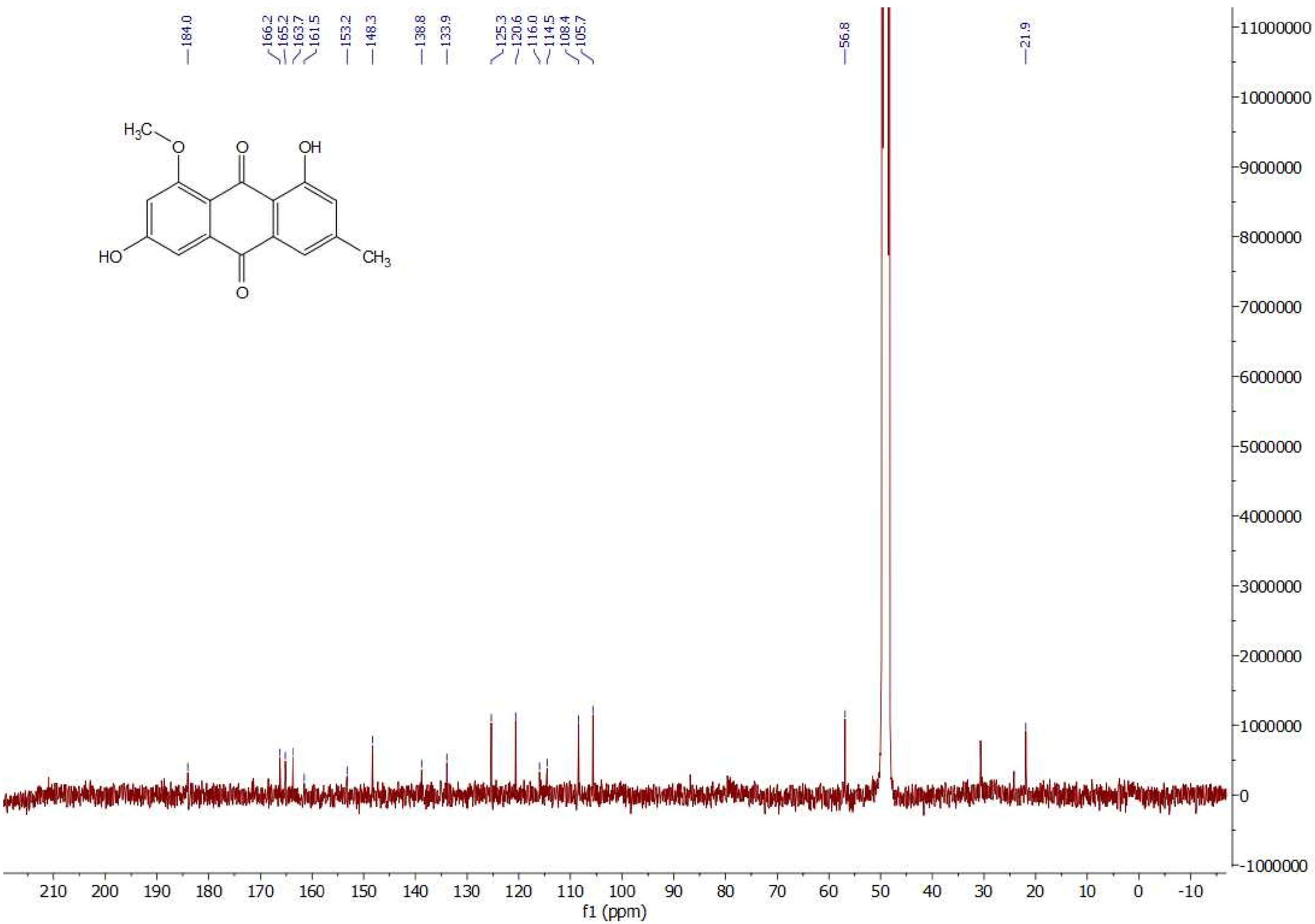

Figure S23: ^{13}C NMR spectrum of compound **4** recorded in MeOD-*d*4 at 100 MHz.

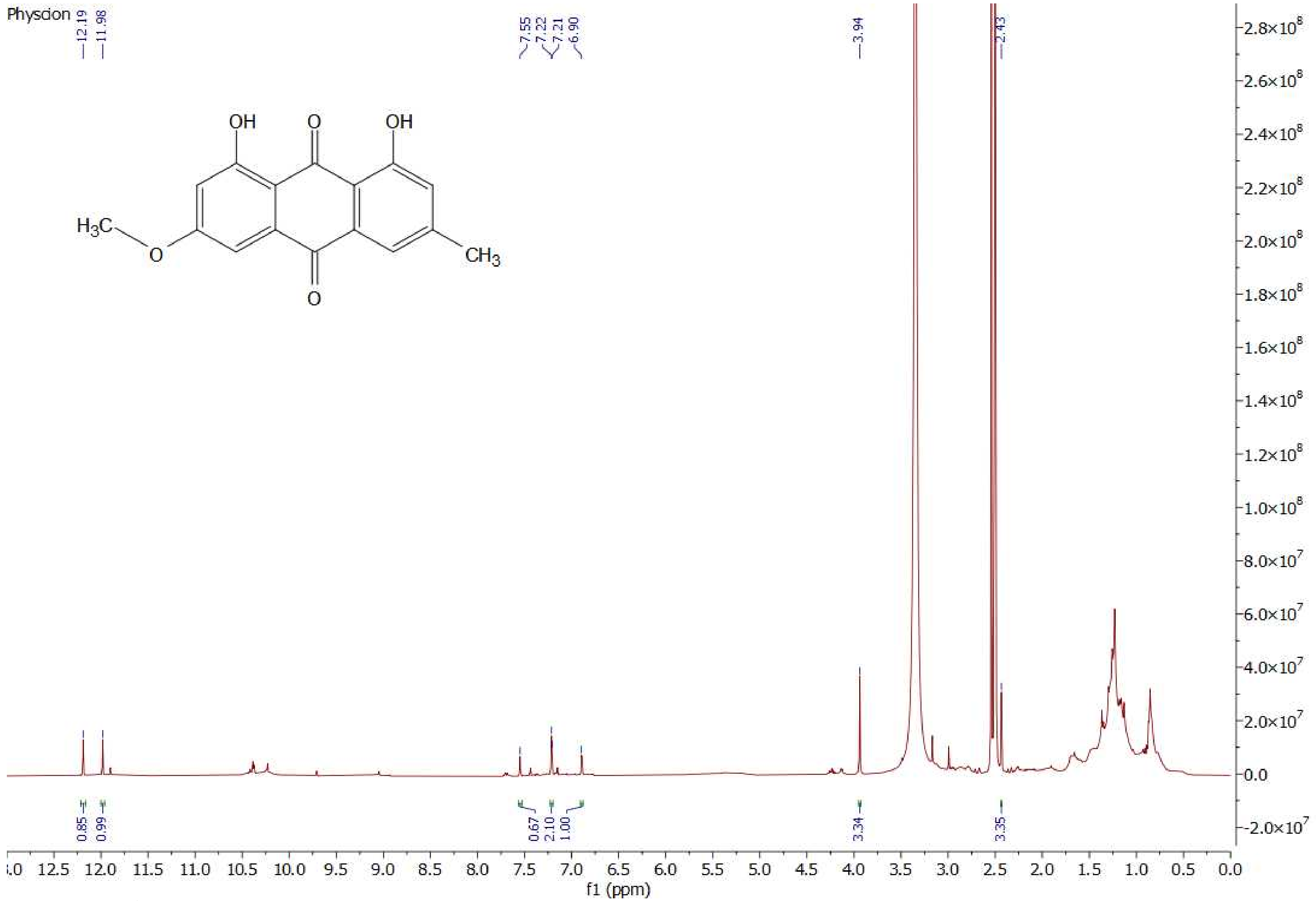

Figure S24: ^{1}HNMR spectrum of compound **5** recorded in DMSO-d_6 at 400 MHz.

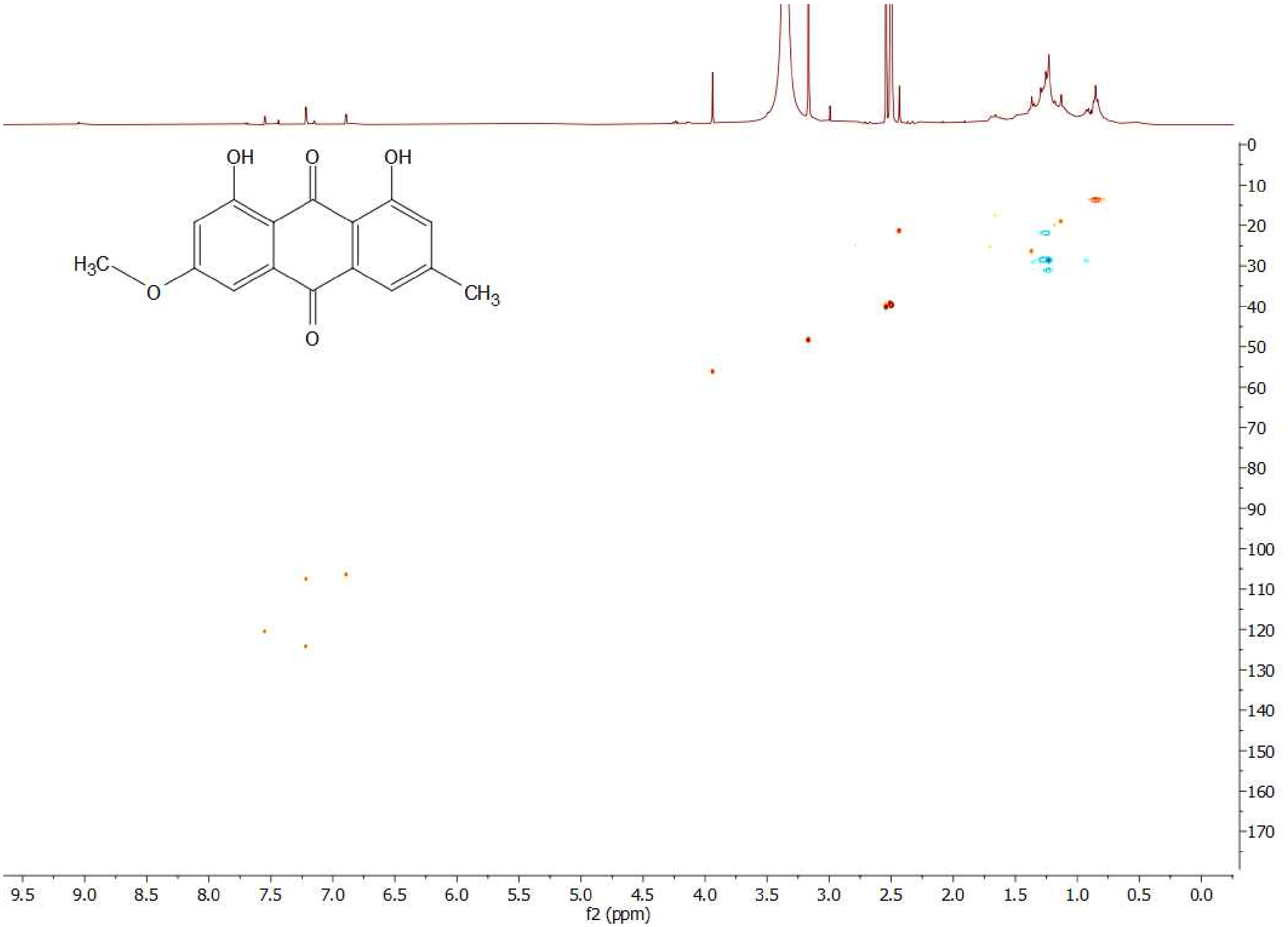

Figure S25: HSQC-DEPT spectrum of compound **5** recorded in DMSO-d_6 at 400 MHz

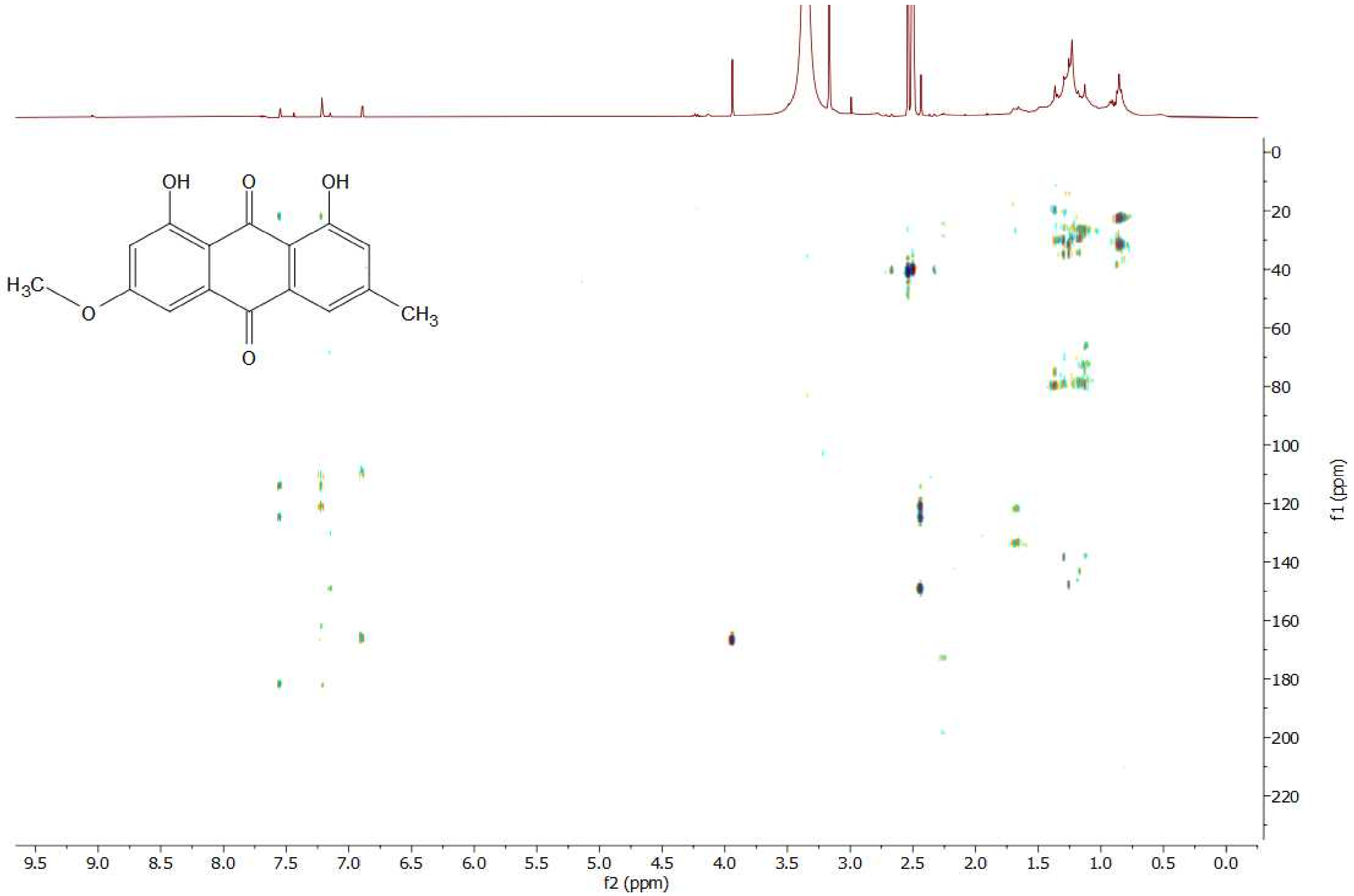

Figure S26: HMBC spectrum of compound **5** recorded in DMSO-d_6 at 400 MHz

Figure S27: Calculated mean torsion angles Φ and ^{3}J (Hz) for H1-H2, H2-H3 and H3-H4 of all 20 E and T conformers of the two enantiomers of Ochraceopyronide **1**.

β-L-Ochraceopyronide	Torsion *Φ*																			
	$^{3}T_{2}$	^{3}E	$^{3}T_{4}$	E_{4}	$^{0}T_{4}$	^{0}E	$^{0}T_{1}$	E_{1}	$^{2}T_{1}$	^{2}E	$^{2}T_{3}$	E_{3}	$^{4}T_{3}$	^{4}E	$^{4}T_{0}$	E_{0}	$^{1}T_{0}$	^{1}E	$^{1}T_{2}$	E_{2}
H1-H2	-34	-14	-20	0	14	25	-20	45	33	27	38	26	12	6	-7	-30	-26	-35	-32	-49
H2-H3	33	27	38	28	15	-6.5	-7	-30	-34	-35	-32	-49	-30	-14	-20	0	6	25	18	45
H3-H4	-30	-35	-32	-49	-31	-14	18	0	14	25	18	45	33	27	36	30	12	-6.5	-7	-30
	^{3}J (Hz)																			
	$^{3}T_{2}$	^{3}E	$^{3}T_{4}$	E_{4}	$^{0}T_{4}$	^{0}E	$^{0}T_{1}$	E_{1}	$^{2}T_{1}$	^{2}E	$^{2}T_{3}$	E_{3}	$^{4}T_{3}$	^{4}E	$^{4}T_{0}$	E_{0}	$^{1}T_{0}$	^{1}E	$^{1}T_{2}$	E_{2}
H1-H2	3.5	6.5	5.8	8.1	8.7	8.4	5.8	6.5	7.6	8.1	7.1	8.4	8.8	8.5	7.7	4.3	5.06	3.5	4.3	1.4
H2-H3	5.48	6.6	4.8	6.1	7.5	7.9	7.9	6.1	5.48	5.48	6.1	3.5	6.1	7.48	7.1	7.8	7.48	6.6	7.1	4.18
H3-H4	7.8	7.3	7.8	5.4	7.8	8.4	8.2	2.1	3.4	4.9	2.7	4.96	6.9	8.2	8.2	7.8	8.13	7.36	7.8	5.4
β-D-Ochraceopyronide	Torsion *Φ*																			
	$^{3}T_{2}$	^{3}E	$^{3}T_{4}$	E_{4}	$^{0}T_{4}$	^{0}E	$^{0}T_{1}$	E_{1}	$^{2}T_{1}$	^{2}E	$^{2}T_{3}$	E_{3}	$^{4}T_{3}$	^{4}E	$^{4}T_{0}$	E_{0}	$^{1}T_{0}$	^{1}E	$^{1}T_{2}$	E_{2}
H1-H2	-30	-20	-12	0	12	23	28	46	31	33	29	24	-6	-7	-5	-24	-38	-34	-36	-43
H2-H3	31	33	27	31	14	-7	-5	-24	-38	-34	-36	-43	-9	-12	-20	0	24	23	28	46
H3-H4	-38	-34	-27	-43	-30	-30	-20	0	24	23	28	46	31	33	29	24	-6	-7	-5	-24
	^{3}J (Hz)																			
	$^{3}T_{2}$	^{3}E	$^{3}T_{4}$	E_{4}	$^{0}T_{4}$	^{0}E	$^{0}T_{1}$	E_{1}	$^{2}T_{1}$	^{2}E	$^{2}T_{3}$	E_{3}	$^{4}T_{3}$	^{4}E	$^{4}T_{0}$	E_{0}	$^{1}T_{0}$	^{1}E	$^{1}T_{2}$	E_{2}
H1-H2	8.1	8.5	8.6	8.1	7.15	5	4.3	2	4.3	3.5	4.3	5	8.4	8.4	8.4	8.4	7.1	7.6	7.6	6.4
H2-H3	6	5.48	6	6	7.7	7.9	7.9	6.6	4.8	5.48	5.48	4.18	7.7	7.7	7.1	8	6.6	6.6	6	4.18
H3-H4	2.7	3.4	4	2	4.2	4.2	5.7	7.9	8.1	8.1	7.8	6.2	7.8	7.3	7.8	8.1	7.48	7.48	7.5	4.96

Figure S28: Theoretical $^{3}J_{HH}$ coupling constants for each of the three pairs H1-H2, H2-H3, H3-H4, for the dihedral angle range -60 to 60, based on the Altona equation.

β-D-C-lyxofuranoside residue	^{3}J (Hz)			*β*-L-C-lyxofuranoside residue	^{3}J (Hz)		
Torsion angles	H1-H2	H2-H3	H3-H4	Torsion angles	H1-H2	H2-H3	H3-H4
-60	4.28	2.32	0.499	-60	0.47	2.3	4
-55	5.03	2.9	0.92	-55	0.89	2.9	4.8
-50	5.77	3.53	1.44	-50	1.4	3.5	5.4
-45	6.46	4.18	2.06	-45	2.06	4.18	6.2
-40	7.1	4.84	2.74	-40	2.8	4.8	6.7
-35	7.65	5.48	3.46	-35	3.5	5.48	7.36
-30	8.1	6.08	4.213	-30	4.3	6.1	7.8
-25	8.436	6.6	4.96	-25	5.06	6.6	8.13
-20	8.65	7.1	5.68	-20	5.8	7.1	8.2
-15	8.72	7.48	6.35	-15	6.5	7.48	8.43
-10	8.66	7.77	6.97	-10	7.15	7.7	8.3
-5	8.46	7.9	7.48	-5	7.7	7.9	8.2
0	8.146	8	7.9	0	8.1	8	7.8
5	7.7	7.94	8.2	5	8.47	7.94	7.48
10	7.158	7.77	8.38	10	8.8	7.8	6.9
15	6.5	7.48	8.43	15	8.7	7.48	6.35
20	5.818	7.1	8.34	20	8.6	7.1	5.6
25	5	6.6	8.13	25	8.4	6.6	4.96
30	4.29	6.08	7.8	30	8.1	6.1	4.2
35	3.5	5.48	7.36	35	7.6	5.48	3.46
40	2.768	4.84	6.821	40	7.1	4.8	2.7
45	2.06	4.18	6.2	45	6.46	4.18	2.06
50	1.437	3.55	5.53	50	5.77	3.5	1.5
55	0.89	2.9	4.816	55	5	2.9	0.92
60	0.47	2.3	4.1	60	4.3	2.37	0.5

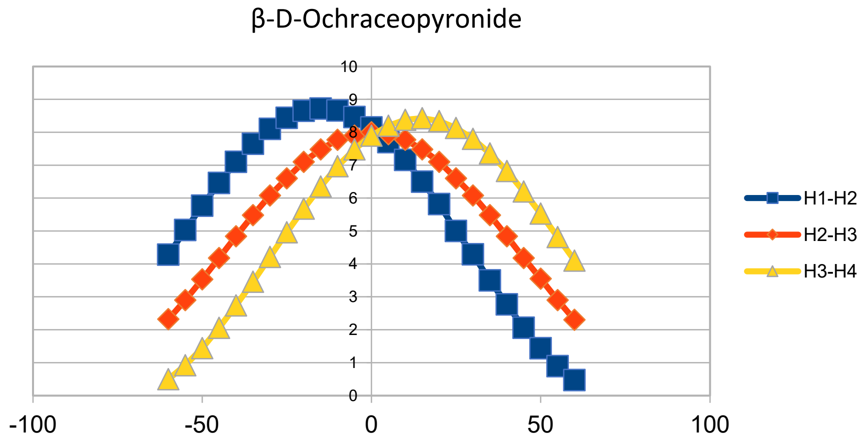

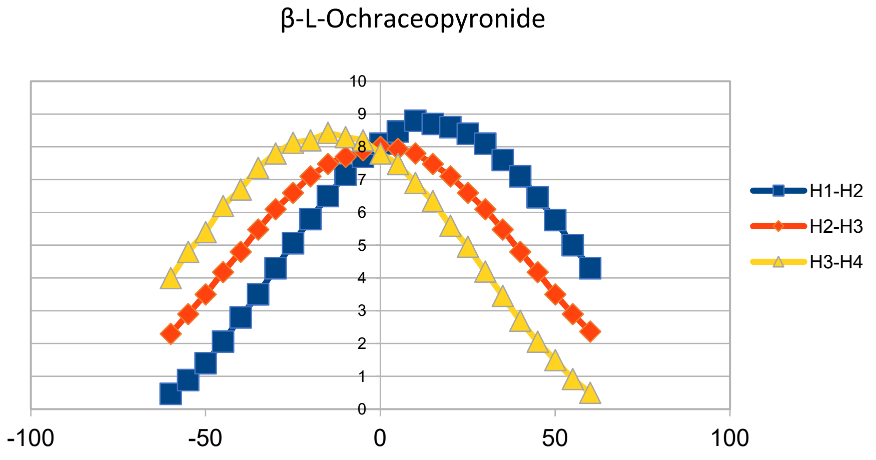

Figure S29: Distribution of furanose conformers of β-D- and β-L-C-lyxofuranoside residues of Ochraceopyronide (**1**), after Molecular Dynamics simulation.

Conformer	β-D-Ochraceopyronide	β-L-Ochraceopyronide
E_0	0	1.13043
^0E	1.28714	0
E_1	6.04318	0
^1E	0	5.64357
E_2	0.054772	25.0792
^2E	25.0582	0.0770746
E_3	1.10457	4.85142
^3E	5.21703	1.04907
E_4	11.954	0
^4E	0.00456433	12.3491
$^2T_3$	7.15688	0.834975
$^3T_2$	0.899174	7.14225
$^4T_3$	0.0730293	13.9162
$^3T_4$	13.2046	0.0513831
$^4T_0$	0	3.19003
$^0T_4$	3.46889	0
$^1T_0$	0	1.97397
$^0T_1$	2.10872	0
$^1T_2$	0	22.7113
$^2T_1$	22.3652	0

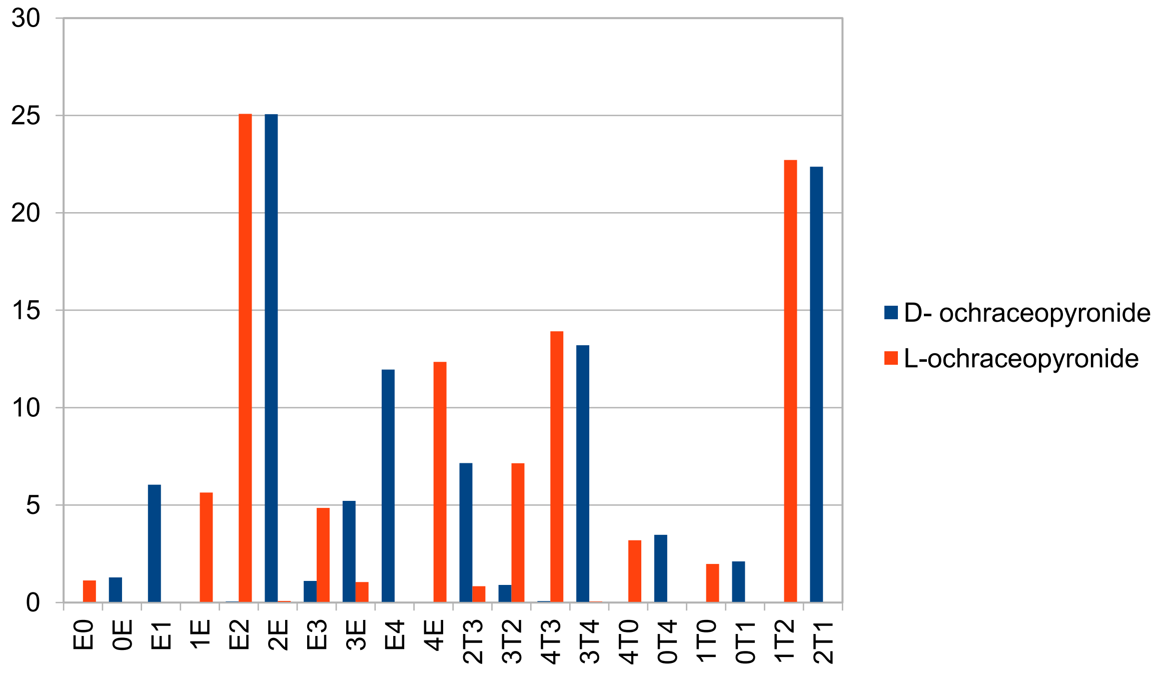

Figure S30: Pseudorotation wheel for Ochraceopyronide.

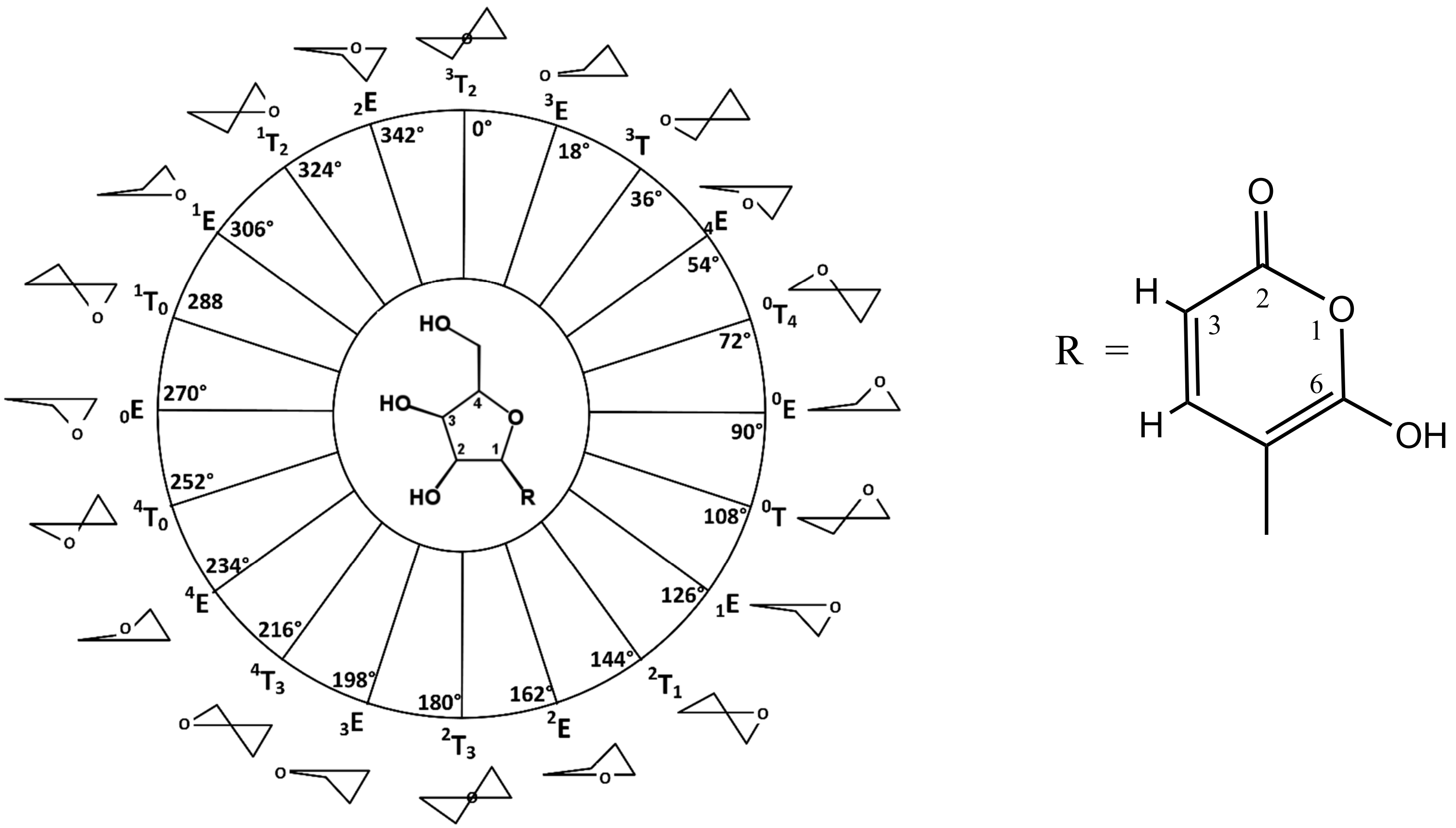

Supplement: Supplementary file 1 [file molecules-26-03976-s001.zip › molecules-1250891-supplementary.pdf]
